# Dynamic mode decomposition for analysis and prediction of metabolic oscillations from time-lapse imaging of cellular autofluorescence

**DOI:** 10.1038/s41598-025-07255-4

**Published:** 2025-07-02

**Authors:** Daniel Wüstner, Henrik Helge Gundestrup, Katja Thaysen

**Affiliations:** Department of Biochemistry and Molecular Biology, University of Southern Denmark, Campusvej 55, Odense M, 5230 Denmark

**Keywords:** Biochemistry, Biological techniques, Biophysics, Computational biology and bioinformatics, Microbiology, Biomarkers

## Abstract

Oscillations are a common phenomenon in cell biology. They are based on non-linear coupling of biochemical reactions and can show rich dynamic behavior as found in, for example, glycolysis of yeast cells. Here, we show that dynamic mode decomposition (DMD), a numerical algorithm for linear approximation of non-linear dynamics, can be combined with time-delay embedding (TDE) to dissect damped and sustained glycolytic oscillations in simulations and experiments in a fully data-driven manner. Together with an assessment of spurious eigenvalues via residual DMD, this provides a unique spectrum for each scenario, allowing for high-fidelity time-series and image reconstruction. By machine-learning-based clustering of identified DMD modes, we are able to classify NADH oscillations, thereby discovering subtle phenotypes and accounting for cell-to-cell heterogeneity in metabolic activity. This is demonstrated for varying glucose influx and for yeast cells lacking the sterol transporters Ncr1 and Npc2, a model for Niemann Pick type C disease in humans. DMD with TDE can also discern other types of oscillations, as demonstrated for simulated calcium traces, and its forecasting ability is on par with that of Long Short-Term Memory (LSTM) neural networks. Our results demonstrate the potential of DMD for analysis of oscillatory dynamics at the single-cell level.

**Supplementary Information:**

The online version contains supplementary material available at 10.1038/s41598-025-07255-4.

## Introduction

The energy metabolism of eukaryotic cells is tightly controlled to meet the demands of a constantly changing environment. Oscillations in abundance of metabolites are a hallmark of cellular respiration in various cell types^1,2^. They have been studied in great detail in baker’s yeast, *Saccharomyces cerevisiae*, in which oscillations occur due to fluctuations in concentrations of key metabolites, accompanied by oscillations in mitochondrial membrane potential^3–5^. Baker’s yeast uses glucose as the primary energy source during exponential growth, and a large body of work has been devoted to the analysis of glycolytic oscillations in this model organism^6^.

Glycolytic oscillations can be monitored by label-free measurements of the intrinsic fluorescence of the reduced form of the electron carriers nicotinamide adenine dinucleotide (phosphate) (NAD(P)H) with an excitation around 360 nm and emission between 400 and 500 nm^5,7^. Once oxidized to NAD(P)^+^, the fluorescence signal vanishes, while reformation of the reduced form, NAD(P)H, gives rise to a new fluorescent signal. NAD(P)H can also be excited at 720 nm by a two-photon process, which is particularly suitable for mammalian cells and tissues, whose thickness prevents high-contrast imaging by one-photon excitation^8–10^. While nicotinamide adenine dinucleotide phosphate (NADPH) is the main electron carrier in anabolic reactions, nicotinamide adenine dinucleotide (NADH) serves the same role in catabolic processes, such as glycolysis. Thus, oscillations in autofluorescence around 450 nm monitor the interconversion of this electron carrier between the reduced and oxidized state.

In synchronized yeast populations, time-varying NADH concentrations of individual cells oscillate in-phase, allowing one to measure glycolytic oscillations spectrophotometrically^4,5^. In contrast, imaging-based analysis allows for monitoring individual cells, thereby revealing partial coherence phenomena as well as desynchronization dynamics and even glycolytic waves traveling throughout the imaged cell population^11–13^. Various computational methods have been applied to the analysis of such time-lapse video data^14,15^, but such tools are often designed for a particular condition only^11,13,16^.

Here, we present the application of dynamic mode decomposition (DMD) to the analysis of glycolytic oscillations in yeast cells based on time-lapse imaging of cellular autofluorescence of NADH fluctuations. DMD is a projection-based computational technique providing a linear approximation of non-linear dynamics in a purely data-driven manner^17^. DMD aims to describe complex spatiotemporal signals by a finite-dimensional approximation of the Koopman operator using only measured snapshots of the dynamics as input. This allows for approximating non-linear dynamics by a linear matrix decomposition, allowing dynamic modes to be separated by the individual time scales of the underlying dynamics.

DMD has been widely applied to imaging data, including separation of background from foreground, object tracking, video shot detection, motion correction, image segmentation, disease diagnostics in biomedical imaging and detection of intracellular protein aggregates from live-cell microscopy data^18–24^. By combining DMD with time delay embedding (TDE), one can apply time-shifted versions of the original data, thereby enriching the data with past information to improve the reconstruction capabilities^17,25–27^. This method is based on Taken’s embedding theorem, which states that for a given time series sampled from a high-dimensional attractor, one can approximate the attractor using time-shifted versions of the original time series^27,28^. This approach has been implemented in several variants of DMD, including higher-order DMD (HoDMD) and Hankel alternative view of Koopman (HAVOK) analysis, for example, for analysis of fluid dynamics and for reconstructing 3D fluorescence microscopy data^25,26,29,30^.

Here we show that DMD with TDE allows for accurate reconstruction of simulated and experimental glycolytic oscillations, and we assess the extent of time delay needed to achieve faithful results. We show that DMD’s ability to predict future time points depends on the delay-embedding dimension and is on par with that of Long-Short-Term-Memory (LSTM) networks. Finally, we combine DMD with TDE and feature-based clustering to classify single-cell trajectories for varying glucose influx and in a yeast model of Niemann Pick type C (NPC) disease. Together, our results show the power of DMD and provide a guide in using this method for analysis of biological time series data gathered by time-lapse imaging of living cells.

## Results

### Damped and sustained oscillations in a simple model of Glycolysis

We aimed to simulate realistic glycolytic oscillations in a population of cells, which can serve as input for the DMD analysis described below. For the sake of our analysis, we restrict the simulations of glycolysis to a minimal model, which produces damped and sustained oscillations in dependence of interpretable model parameters.

**Fig. 1:**
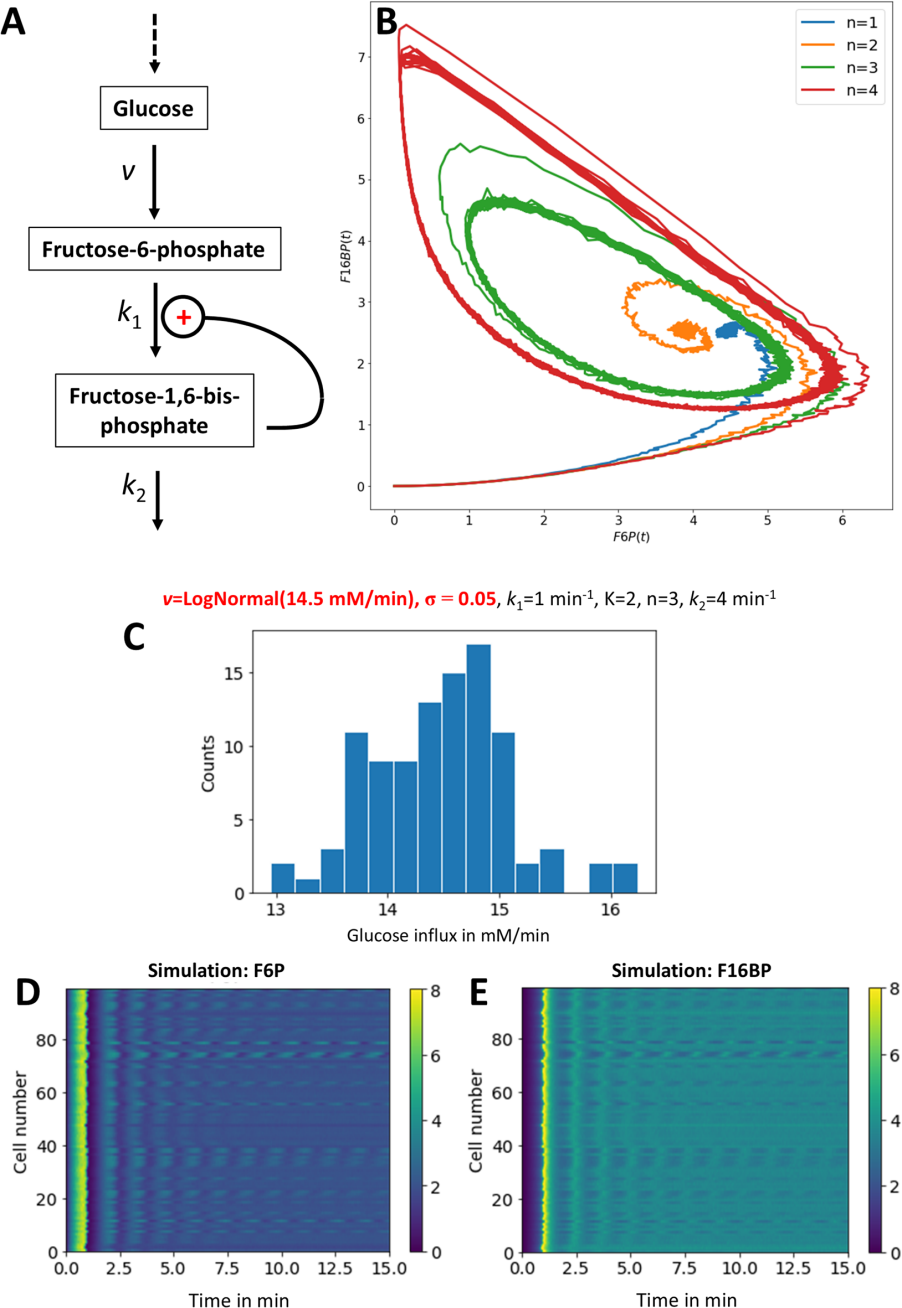
Minimal model to simulate cell-to-cell variability of glycolytic oscillations. A steady state model of glycolysis has been formulated based on positive allosteric regulation of phosphofructokinase by its product fructose-1-6-bisphosphate (F16BP). In this model, glucose is supplied with constant influx v, while the concentrations of fructose-6-phosphate (F6P) and of F16BP are allowed to vary (**A**). A positive feedback indicated by ‘+’ stimulates the conversion of F6P to F16BP followed by constant reaction flux, removing F16BP during the following steps of glycolysis. This model produces different phase portraits depending on the extent of cooperativity of the feedback loop, as shown in panel (**B**); damped oscillations for *n* = 1 and 2 but a limit cycle with sustained oscillations for high cooperativity, i.e., *n* = 3 and 4. (**C**–**E**) A distribution of 100 influx values was generated from a log-normal distribution centered around 14.5 mM/min (**C**). Simulation of time courses for F6P (**D**) and F16BP (**E**) using these influx values and the other indicated parameters (see top of panel **C**).

Our model is a version of the one developed by Higgins (1964) and is based on positive feedback due to allosteric activation of phosphofructokinase (PFK) by fructose-1,6-bisphosphate (F16BP)^3,31^. This can be modeled by a simple autocatalytic reaction network of the form shown in Fig. 1A, in which we consider an autocatalytic mechanism of oscillations of two glycolytic intermediates, fructose-6 phosphate (F6P) and F16BP (see system equations in Supplementary information, Eqs. S1 and S2). The reaction rate for the first step, *v*, is constant and describes the constant inflow of glucose into the system. This step resembles the import of glucose and its conversion into glucose-6-phosphate by hexokinase in the presence of constant ATP concentration^32^.

In our toy model of allosteric activation of PFK, the conversion of F6P into F16BP is described by k_1_, the rate constant for the PFK reaction, *n* is the Hill coefficient describing the extent of cooperativity of allosteric activation of PFK by F6P and K is the equilibrium constant for this binding step (see Supplementary information, Eqs. S1 and S2). The second reaction is modeled as a linearized Michaelis-Menten reaction, in which k_2_ is the rate constant for the aldolase reaction. This system can give rise to damped oscillations for *n* = 2 and sustained oscillations for *n* ≥ 3 and for *k*_2_ = 4 min^−1^ (Fig. S1). This is also illustrated in the phase portrait of the two reactions in Fig. 1B, for which the system of Eq. 1A and B were simulated with noise drawn from a log-normal distribution. The latter emulates multiplicative noise due to low-copy number of glycolytic enzymes working under non-saturated conditions in a network with cooperative feedback (Fig. 1B, see Materials and methods for details)^33^. Based on simulations of the model (Fig. S1), we find that both damped and sustained oscillations can form, depending on the chosen parameter values. This is in line with conclusions for the original Higgins model^3^.

While our model is a strong simplification of real yeast glycolysis, it allows us to simulate the key characteristics of glycolysis with a minimal number of parameters. We do not claim that the mechanisms used here are the ones that actually cause glycolytic oscillations in yeast cells. Later studies showed that the oscillations are a consequence of concentration fluctuations of various metabolites, including NADH/NAD^+^, the ATP/AMP ratio and acetaldehyde^34–36^. Indeed, theoretical studies have shown that complex and detailed mechanistic models of yeast glycolysis can be reduced to three key variables, namely ATP, F6P and F16BP^37,38^, but only the latter two are needed to obtain oscillations^3,32,39^.

### DMD with delay embedding reconstructs simulated glycolytic oscillations

To capture non-linear phenomena, the standard DMD method is extended to include past information, and one popular approach for that is to approximate higher-order derivatives of the data by using time-shifted versions of the original data^25^. First, we applied this method, called HoDMD as implemented in the PyDMD Python package, to the simulated glycolytic oscillations^40^. We find that HoDMD can faithfully reconstruct damped and sustained oscillations for varying glucose influx and extent of non-linearity (Fig. S1, red lines). The computed eigenvalues shown on the unit circle for the first species (i.e., F6P) reveal an increasing contribution of imaginary values as the system becomes more oscillatory due to higher values of the feedback cooperativity, *n* (Fig. S2A-D). A very similar behavior was found for the eigenvalues of the second species (i.e., F16BP) with HoDMD, while standard DMD failed to dissect the dynamics of the system properly (not shown). We also find that the HoDMD approach requires high embedding dimensions (*d* = 300) for good reconstruction. Additionally, we obtained efficient smoothing of the time traces at much higher accuracy than by Fourier filtering, particularly for damped oscillations (Fig. S2E-G).

Instead of applying HoDMD, one can augment the snapshot matrix with time-shifted versions of the time series data as consecutive rows forming a so-called Hankel matrix as input for DMD with TDE^29,41,42^. TDE has been extensively used for state-space reconstruction, since it allows for characterizing the dynamic system, even if only partial measurements of the state-space are available^43,44^. This concept can be illustrated by plotting the time course of F6P for a given parameter set against its time-shifted version, where one finds that the attractor of Fig. 1B can be approximated depending on the chosen time-shift *d* (Fig. S3). The approximated attractor, sometimes also called a ‘shadow manifold’, M’, is diffeomorphic to the original manifold, M, but only for certain values of the delay, *d*^43–45^.

Before testing DMD with TDE on the glycolysis model, we determined its behavior for varying parameters. By carrying out a stability analysis of the glycolysis model (see Appendix and Supplementary information), one finds that exponential decays, damped oscillations and limit cycles (sustained oscillations) can form at steady state in dependence of *v*, the influx of glucose (Fig. S4). In addition, varying the feedback cooperativity, *n*, (Figs. S1 and S2) as well as the rate constant, *k*_2_, gives rise to this behavior (not shown, but see^3,32,39^). We focused on varying *v* systematically and applied DMD with TDE to each model output. We found that the high reconstruction quality obtainable by DMD with TDE persists over the entire parameter range of the model (Fig. S5).

### DMD with TDE can reconstruct cell-to-cell variations in metabolic activity

Cell-to-cell variations in metabolic activity can occur in each time-lapse recoding, for example due to varying glucose influx into glycolysis as a result of stochastic expression of the glucose importers in individual cells. Indeed, the genome of *S. cerevisisae* codes for at least twenty isoforms of the hexose transporters (*HXT* genes), which are expressed differently depending on metabolic demands and environmental conditions. Also, inhibition of glucose import with maltose as a competitive inhibitor reduced the frequency of glycolytic oscillations, supporting that the extent of glucose influx is an important control point for metabolic activity^46^. Similarly, varying levels of the hexokinase, which phosphorylates and thereby traps glucose in cells, can give rise to cell-to-cell variations in NADH fluorescence dynamics. In fact, knockout of hexokinase 2, which is responsible for about 50% of glucose phosphorylation in *S. cerevisiae*, reduced the amplitude of NADH oscillations^47^. Furthermore, single-cell studies have shown that cell-to-cell variability in expression of *HEX* genes, coding for yeast hexokinases, can cause varying metabolic activity between individual cells^48^.

To model the effect of cell-to-cell variation in glycolytic activity, we carried out repeated simulations of the reaction system for stochastically varying influx rate *v* centered around *v* = 14.5 mM/min (Fig. 1C). In this range of glucose influx varying between ca. 12.5 and 16.5 mM/min, a stable focus characterized by damped oscillations will form for most of the simulated cells. However, occasional sustained oscillations are also found when the glucose influx is around 12.5 mM, and as the data is arranged, such time traces are located next to those with damped oscillations (compare Fig. 1D, E). These simulations not only support the central role of glucose influx on the oscillatory behavior of the model, as described previously^3,38^. They also enable us to assess the ability of DMD to dissect single-cell time series of metabolic activity from an array of cells, as described below.

**Fig. 2:**
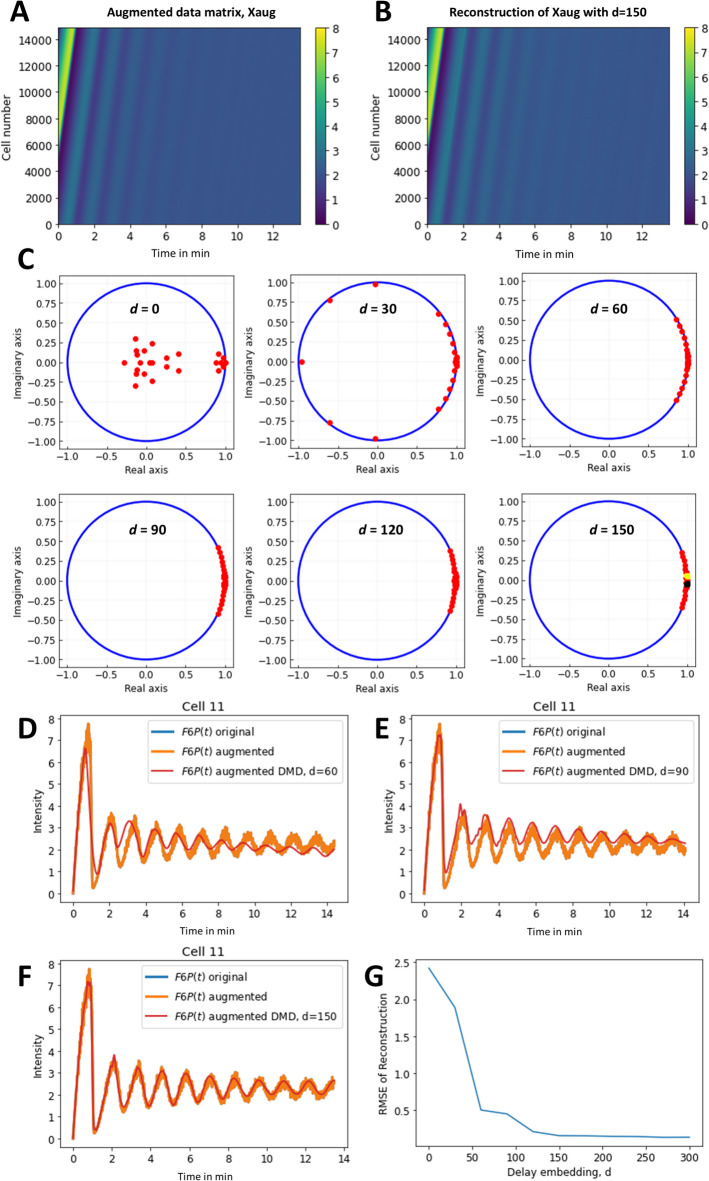
Generation of Hankel matrix and its analysis using DMD for varying delays. Time-delay embedding was realized by generating Hankel matrices of the simulations of the first species, i.e., F6P, as shown in Fig. 1D. For that, time-shifted versions of each simulated oscillation were generated and stacked as additional rows to the original data matrix, giving an augmented data matrix (**A**). Applying DMD to this augmented matrix results in very good reconstructions (**B**). Eigenvalue spectrum of the identified DMD modes for varying extent of delay embedding (**C**). For increasing *d*, the eigenvalues shift to the right and concentrate around the ones determined for the simulated system (yellow and black dots on plot for *d* = 150, see Eq. A8 and A9). (**D**–**F**) simulated time courses for a selected cell (Cell 11), with original data from matrix X in blue, data from the augmented matrix Xaug in orange (which is identical to those of matrix X).

To apply DMD to this data, we first generated an augmented version of this cell-time matrix using time-shifted versions of the simulated time courses. This was achieved by stacking *d* shifted versions of each row (resembling a single time series for one value of the glucose influx) consecutively to obtain the augmented matrix (Xaug, Fig. 2A).

Applying DMD to this augmented matrix results in very accurate reconstruction of the data (Fig. 2B). This is even the case for a low-rank approximation (i.e., *r* = 25) but only as long as the extent of delay embedding is high enough (Fig. 2C–F). A systematic analysis of the extent of TDE reveals that the eigenvalue spectrum becomes stable for delays *d* > 120, coinciding with good reconstruction quality for the damped oscillations for each cell (Fig. 2C–G). In fact, the reconstruction quality converges towards low RMSE values above *d* = 150 (Fig. 2G).

For comparison, we calculated the analytical eigenvalues for the same data set, rescaled them to the unit circle and plotted them on top of the eigenvalue spectrum for d = 150 (Fig. 2C, last panel, yellow and black dots). This comparison shows that the eigenvalues determined by DMD concentrate around the analytical eigenvalues for increasing delay embedding, while at the same time, the reconstruction quality increases. Since the analytical eigenvalues are derived from the system linearized around the steady state (see Appendix), this important result confirms that DMD achieves an optimal linear approximation of the non-linear dynamic system^17,49^. Note that there are 200 analytical eigenvalues for the entire simulated data set of 100 cells, i.e. 2 conjugated eigenvalues for each cell. In contrast, the DMD approximation only contains 25 eigenvalues for the entire data set due to the low-rank truncation of the Koopman operator with the time-delayed snapshots as observables^42^.

**Fig. 3:**
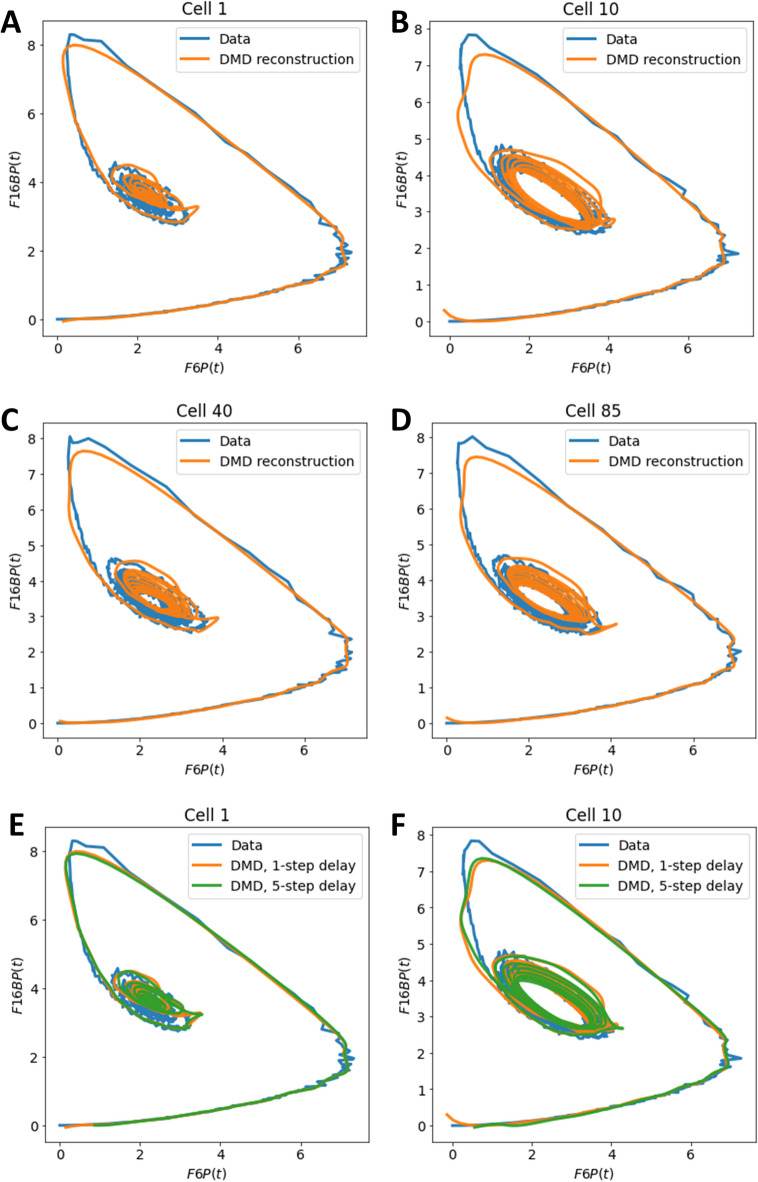
Reconstruction of the system attractor using DMD with time-delay embedding. The two-state glycolysis model was simulated with added noise and analyzed using DMD as described in the legends to Figs. 3 and 4. The time course of the two metabolites, F6P and F16BP, was plotted against each other for selected cells of the simulation with stochastic glucose input, which are indicated on top of each panel (blue lines). The corresponding reconstructions of the time courses are plotted for a 1-step delay in orange and for a 5-step delay in blue for the same cells. Cell 1 is shown in (**A**) and (**E**), cell 10 in (**B**) and (**F**), cell 40 in (**C**) and cell 85 in (**D**).

In the reconstruction, each time course is represented by a weighted sum of these 25 eigenvalues and eigenfunctions (see Eqs. 8 and 9 in Materials and methods). Thus, while the state dimension of the input data is increased by delay embedding, the identified dynamic modes span a low-dimensional subspace of the infinite-dimensional Koopman operator, allowing for faithful reconstruction of the observed dynamics. This is nicely illustrated, when looking at the attractor of the simulated system in comparison to the attractor reconstructed by DMD with time-delay embedding (Fig. 3). Both, the attractors of a stable focus and of limit cycles caused by differences in glucose influx for individual cells are correctly reconstructed by DMD (Fig. 3A–D). Moreover, the reconstructed manifold *M*_DMD_ is much smoother than the simulated one, emphasizing the potential of DMD for time series denoising. It has been suggested that changing the delay step length can improve reconstruction quality of the embedding^50^, so we tested this by increasing the step length for the delay embedding from 1 to 5, but this gave almost identical results (Fig. 3E,F).

Together, DMD with TDE is a powerful tool to dissect and reconstruct complex metabolic time series of non-linear biochemical models. This is not only the case at the single-cell level but also for reconstruction of an entire heterogeneous cell population, which is very important for capturing cell-to-cell heterogeneity in metabolic activity from time-lapse video data, as outlined in the next paragraph.

### DMD reconstruction of starvation-induced oscillations of NAD(P)H in yeast cells

**Fig. 4:**
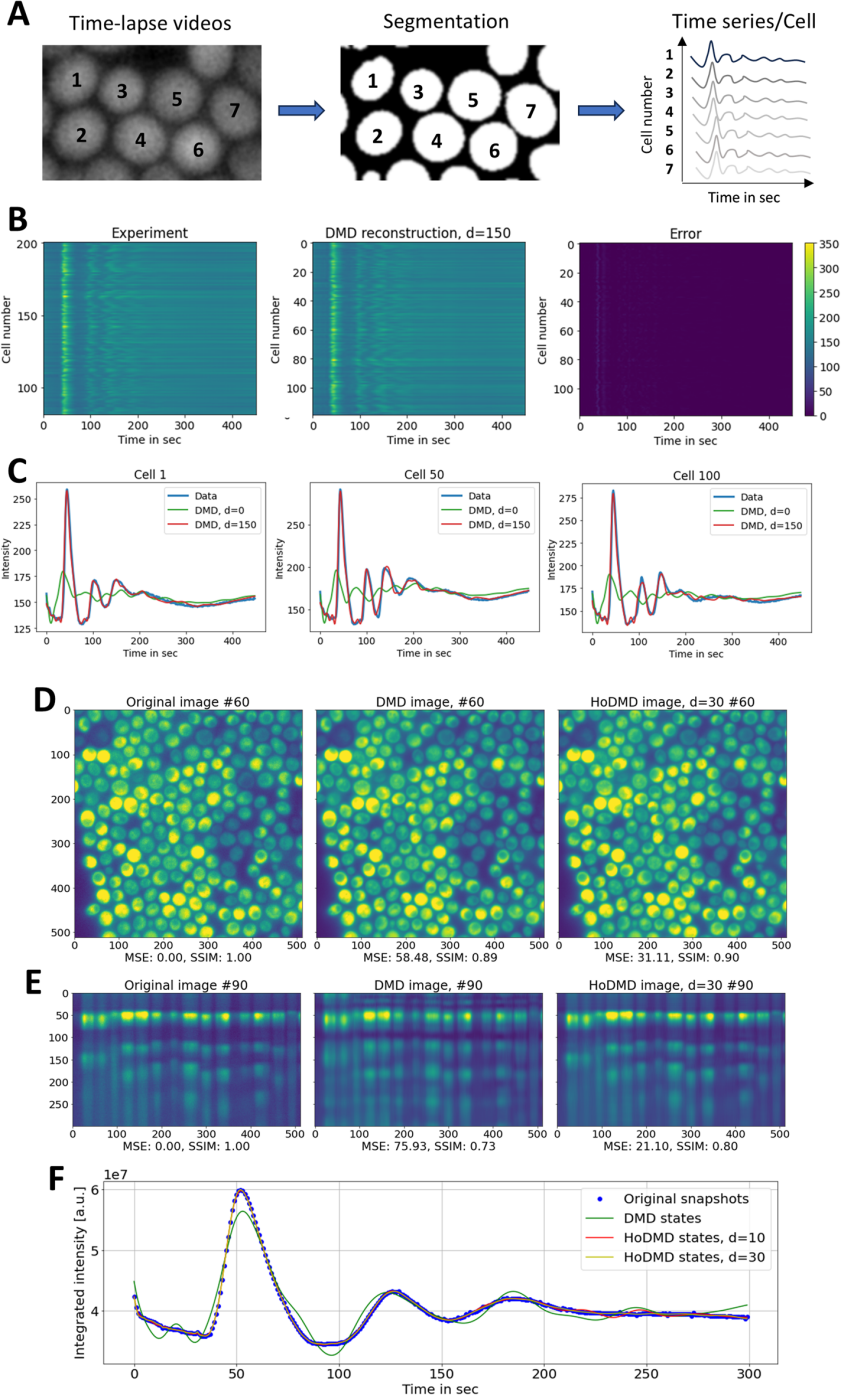
DMD reconstruction of experimental autofluorescence oscillations in yeast cells. Yeast cells were pre-starved, washed and placed on a microscope dish coated with poly-D-lysine before imaging on a wide field microscope as described in Materials and methods. (**A**–**C**) Video recordings were segmented and individual time traces of mean intensities arranged as matrix, exactly as used in the simulations. B, experimental data sets (left panel) were reconstructed using DMD with time-delay embedding of *d* = 150 (middle panel), resulting in a very low reconstruction error (right panel). (**C**) Intensity traces of selected cells from the experimental data (cell number is given on top of each panel) are plotted (blue lines) and compared to the DMD reconstruction without embedding (*d* = 0, green lines) and with embedding (*d* = 150, red lines). (**D**–**F**) HoDMD reconstruction of entire video sequence without segmentation of cells. (**D**) Original time-lapse recording (left panel), DMD reconstruction (without delay embedding, middle panel) and HoDMD reconstruction (with *d* = 30, right panel), all shown for frame #60 of the corresponding image stack. (**E**) The same as (**D**) but side view (xt-view) along the horizontal line #90. Image quality measures, i.e., MSE and SSIM, relative to the original data are given below each panel. F, integrated intensity along the time axis for raw data (blue dots), DMD reconstruction with *d* = 0 (green line), HoDMD with *d* = 10 (red line) and *d* = 30 (yellow line), respectively.

To assess the performance of DMD with delay embedding in reconstructing real metabolic oscillations, we recorded cellular autofluorescence in baker’s yeast *S. cerevisiae*. The intrinsic fluorescence of NADH is a measure of glycolytic flux and has been studied extensively in yeast and other eukaryotic cells (see Introduction). We used a yeast strain, which is unable to rely on oxidative phosphorylation due to defective heme synthesis (i.e., *hem1*Δ cells). When being pre-starved, *hem1Δ* cells respond to addition of glucose with (damped) oscillations of NADH fluorescence (Fig. 4). Time traces of autofluorescence were extracted as mean intensity per cell after segmenting individual cells. For segmentation, we have used a pre-trained deep-learning model, and the mean intensity per cell was afterwards aligned in a cell-time plot, exactly as for the simulations (see Materials and methods and Fig. 4A).

DMD with TDE but not standard DMD reconstructs the time series for each cell accurately, as long as the extent of embedding is sufficient (i.e., *d* = 150; Fig. 4B,C). These results agree with our simulation results (see Fig. 2 and S5), and they are also supported by earlier findings showing that the extent of delay embedding needed for faithful reconstruction of time series depends on the spatial dimension of the data^26,27,29^. In fact, as shown by Mezic and co-workers, the true Koopman eigenvalues and eigenfunctions for an ergodic system can be found from a Hankel matrix in the limit of infinite-time observations^42^. Since the experimental metabolic oscillations are not stationary, it is interesting that we still achieve very good approximation of the data using DMD with time delays.

A potential problem of the above analysis is that a finite-dimensional approximation of the infinite-dimensional Koopman operator can result in spectral pollution, i.e., the occurrence of spurious eigenvalues that are not related to the evolution operator^51^. While the Hankel-matrix-based DMD method theoretically ensures to recover the correct spectrum of the Koopman operator (see above), this is only guaranteed in the limit of infinite observations for an ergodic system^42^. Also, the Koopman operator can have a continuous spectrum, particularly for chaotic systems^51^. To validate the eigenvalues identified by DMD with delay embedding, we re-analyzed the simulated and experimental data sets using residual DMD, a method to identify pseudo-eigenfunctions based on calculations of residuals for each eigenvalue pair^51,52^.

We find for both the simulated and the experimental data that without delay embedding, a portion of the eigenvalues, particularly towards the center of the unit circle, are associated with large residuals, indicating that they are spurious eigenvalues (Fig. S6)^52^. In contrast, with delay embedding (*d* = 150), a larger part of the eigenvalue spectrum is associated with low residuals, showing that more of the calculated basis functions actually contribute to a linearized description of the data. As a result, we achieve a better approximation of the Koopman operator and thereby of the simulated and measured fluorescence dynamics, respectively (Fig. S4, right columns). This result further emphasizes the power of delay embedding as part of the DMD method in dissecting non-linear dynamical systems^26^.

To analyze, not individual cells, but the entire video data at once without prior cell segmentation, we returned to the HoDMD analysis. This is because HoDMD has been shown to be particularly useful for analyzing multi-dimensional time series by taking the history of the data into account^25^. Again, HoDMD but not standard DMD can reconstruct the transient oscillations found in starved yeast cells upon addition of glucose, while standard DMD without TDE was not successful (Fig. 4D–F). A higher time-delay embedding was required to reconstruct the dynamics of non-starved cells compared to starved cells (compare Fig. 4D–F with Fig. S7). This is likely a consequence of the more transient nature of the dynamics in non-starved cells and not related to cross differences in the singular value spectrum of both data sets (Fig. S8). Indeed, it has been shown previously that time series with transients decaying faster than the slowest mode represent challenges for DMD^53^. For both situations, i.e., the autofluorescence dynamics of starved and non-starved cells, the singular values of the snapshot matrix generated from the time-lapse video sequence decay rapidly (Fig. S8). This low-rank structure of the data is a pre-condition for the DMD reconstruction with few modes governing the essential dynamics of the system.

### Prediction of metabolic oscillations by DMD with time-delay embedding

Apart from faithful reconstruction, DMD with TDE can also interpolate missing data points (not shown but see^30^). We therefore wondered whether the method also allows for predicting data. Forecasting future events from existing time series reflects the ability of a model to have learned subtle dependencies in the data. To assess the ability of DMD to predict metabolic oscillations, we focused first on the simulated time courses, which we truncated at selected time points and assessed the ability of DMD to reconstruct the training data and to predict unseen data. We found that DMD with TDE allows for predicting damped oscillations for several periods in the future (Fig. S9A-D). However, the predictions are less accurate for sustained compared to damped oscillations (Fig. S9E-H). The limited capability of DMD to predict future time points in highly non-linear systems is also the outcome of other studies and a consequence of the underlying assumptions, particularly the approximation of non-linear dynamics by a linear and finite matrix *A*^54^.

**Fig. 5:**
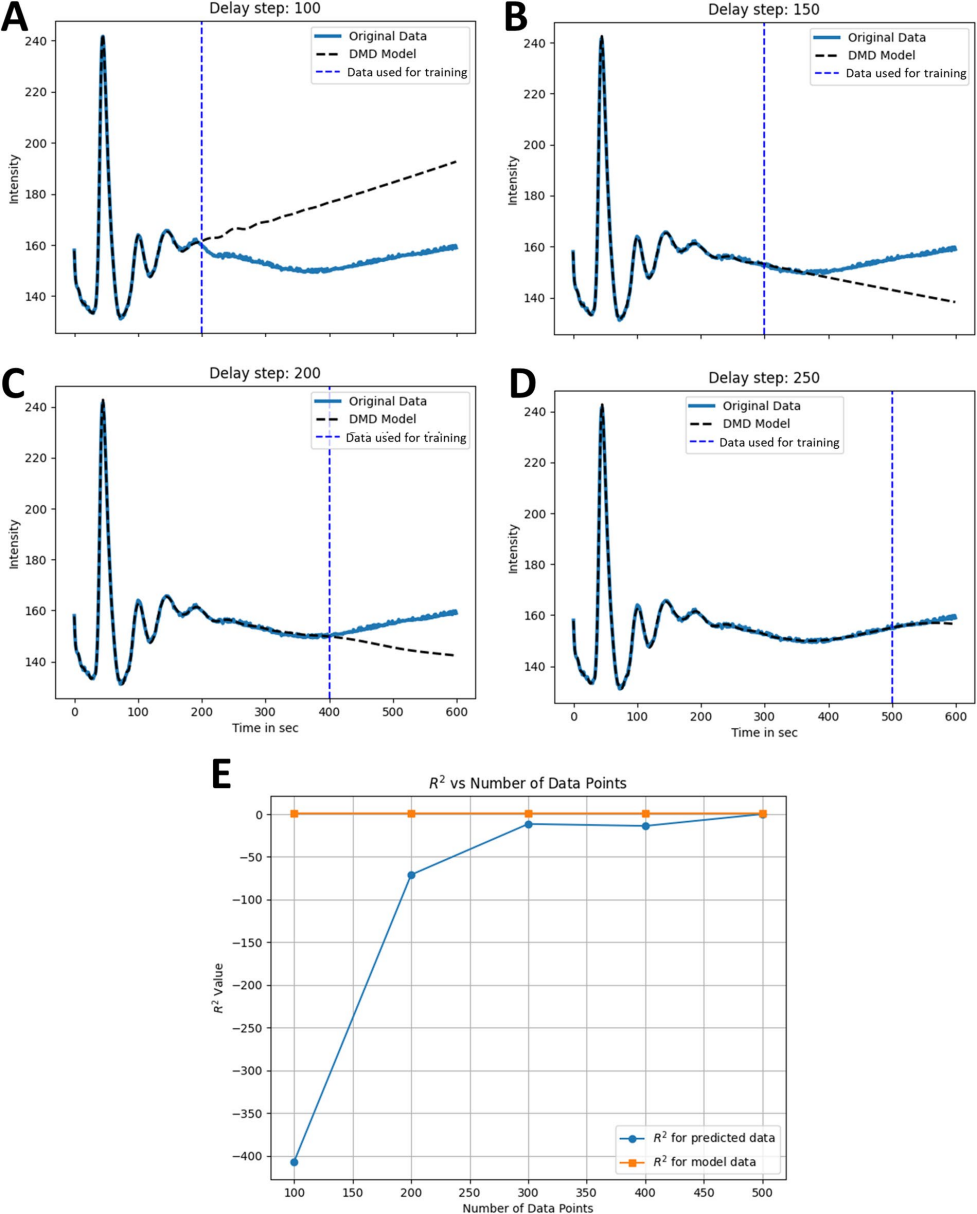
Prediction of experimental time courses using DMD with time-delay embedding. Experimental time courses of NADH fluctuations recorded in response to addition of 15 mM glucose were averaged for all cells in a field (blue lines in **A**–**D**, labeled ‘Original data’) and reconstructed using DMD with time-delay embedding (black dashed lines in **A**–**D**, labeled ‘DMD model’). Training data started at t = 0, and its length was varied with 200 time points in A, 300 in B, 400 in C and 500 time points in (**D**), respectively. This is indicated by the dashed blue line in panel (**A**–**D**) (labeled ‘Data used for training’). (**E**) R^2^-values as function of data points in the training set for the training data (‘model data’, orange line and symbols) and the test data (‘predicted data’, blue line and symbols). See text for further explanations.

Based on these findings, we assessed the ability of DMD with TDE to predict future time points of the experimental autofluorescence time series. For that, experimental data were averaged and split to create the dataset used for training and prediction. Various truncation and delay embedding steps were systematically tested to identify the optimal configuration for capturing the dynamics of the system. Not unexpectedly, DMD performs better with respect to predicting future time points, the more data it has seen, i.e., the deviation from real unseen data becomes smaller for more data points in the training set (Fig. 5 and Fig. S10). Reducing the sampling frequency in the training data by a factor of up to 3 hardly affects the prediction accuracy as long as the time interval for training remains large enough (Fig. S11). The ability to predict future time points was also assessed for HoDMD on entire video sequences. For that, the HoDMD model was trained with 150 out of the 300 image frames.

Despite accurately reconstructing the phase-shifted oscillations of individual yeast cells, HoDMD cannot predict the damped oscillations of cellular autofluorescence apart from the first few frames (Fig. S12). We conclude that the ability of DMD to predict new data points for non-linear time series of glycolytic oscillations is very limited, even after applying TDE. To compare the prediction capability of DMD with delay embedding with other architectures, we re-analyzed the experimental autofluorescence oscillations with a LSTM, a form of recurrent neural networks. LSTMs are designed to prevent exploding or vanishing gradients, when learning long-term dependencies in the time series data^55^. We found that the overall performance of LSTMs to reconstruct and predict the experimental data is not better than that of DMD with delay embedding (Fig. S13 and S14).

### Classification of cell-to-cell heterogeneity in glycolytic oscillations by DMD and machine learning

The metabolic activity can differ tremendously between individual living cells in a population, even when they are of the same genetic background^48,56^. The underlying mechanisms are not fully understood, and an important goal in the emerging realm of single-cell analysis is therefore to characterize this heterogeneity with quantitative tools^56^. In this paragraph, we combine DMD with TDE and data clustering methods to show that time traces of individual cells can be classified based on their unique features captured by DMD.

This is first illustrated for simulated time courses with differing glucose influx, as shown in Fig. S15. For that analysis, we combined three simulated time trace matrices with 100 cells each in which the glucose influx was varied (i.e., *v* = 7 mM/min, *v* = 10 mM/min and *v* = 15 mM/min). Since the DMD amplitudes contain unique information about each cell trace, we calculated those from the identified eigenfunctions. By combining kernel principal component analysis (kernelPCA) of the DMD amplitudes with k-means clustering, we can uniquely identify the different cell populations (Fig. S15A-E). Four clusters based on the first three kernel principal components (PCs) were chosen with cluster 1 and 4 representing the population with sustained oscillations (i.e. *v* = 10 mM/min) and cluster 2 and 3 containing the population of *v* = 15 mM/min and *v* = 7 mM/min, respectively (Fig. S15F). The latter two are characterized by damped oscillations but of differing frequencies and amplitudes, while the population with *v* = 10 mM/min shows a broad range of sustained oscillations (Fig. S15G-J). This is a consequence of being in the unstable region of the parameter space (see Fig. S4 and above), resulting in two scattered clusters. Together, combining DMD with non-linear feature extraction and automated clustering allows for faithful classification of oscillations in simulated cell populations.

**Fig. 6:**
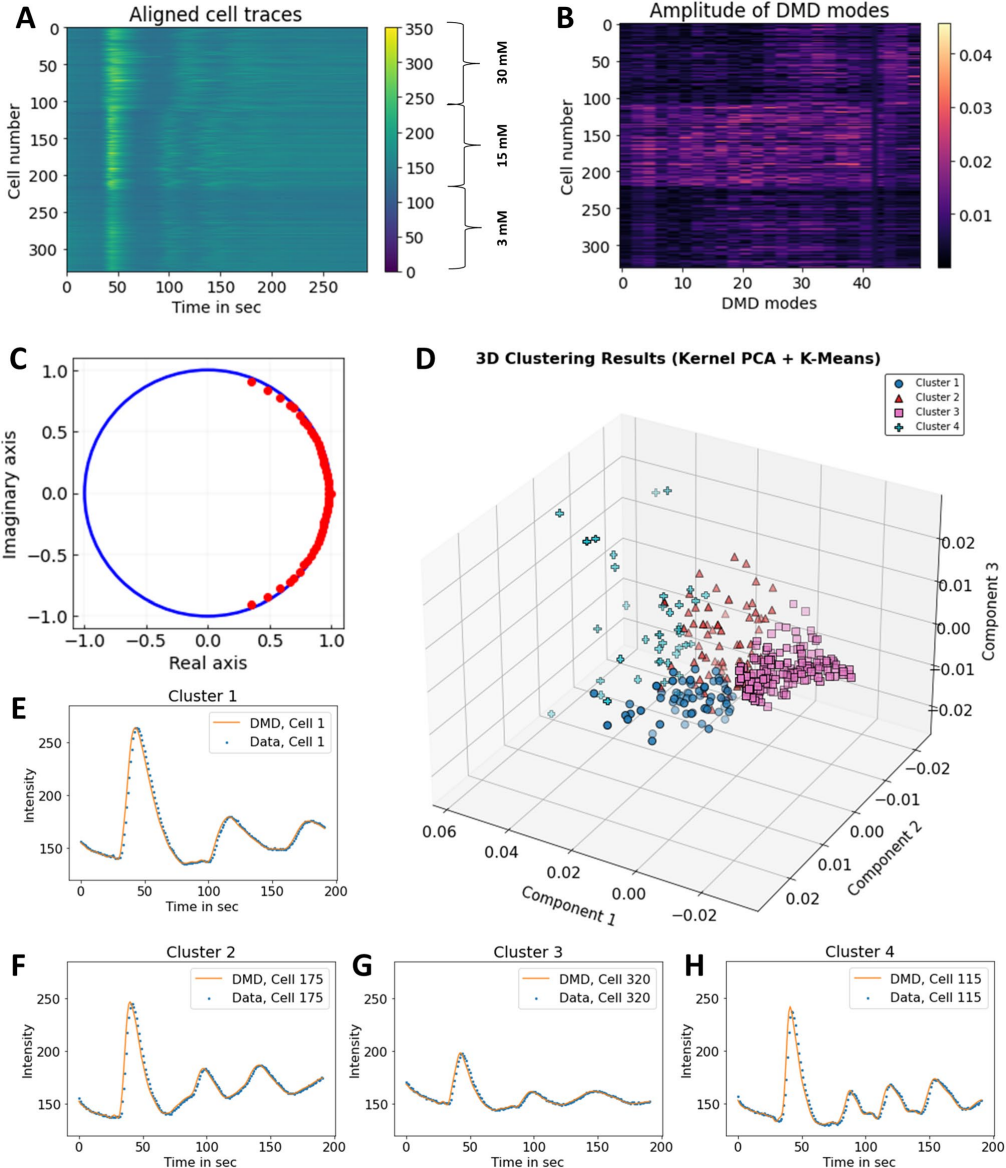
Classification of metabolic oscillations in yeast for varying glucose influx. The *hem1*Δ cells were starved for 3 h in SD-medium without glucose in the absence of dALA before imaging. A bolus of glucose to a final concentration of 30 mM, 15 mM or 3 mM was added after 30 s image recording. Cells were imaged for 300 s in total, intensity traces were extracted after segmentation and arranged as matrix concatenated for all glucose concentrations, (**A**) DMD with delay embedding, *d* = 100 and rank *r* = 50 was applied and mode amplitudes (**B**) and eigenvalues (**C**) were calculated. A kernel PCA was applied to the amplitude matrix, and the first three PCs were clustered into four clusters using k-means clustering, (**D**–**H**) shows example trajectories for each cluster (blue dots, data; orange line, DMD reconstruction).

To validate this approach on experimental data, we repeated the experiment on yeast populations for differing concentrations of glucose and classified them as described above for synthetic data (Fig. 6). At 15 mM applied glucose in pre-starved *hem1Δ* cells, oscillations were most pronounced, which was reflected in unique DMD amplitudes (Fig. 6A, B, middle part, cell 111–220). The matrix *A* was rank-truncated to *r* = 50, resulting in 50 eigenvalues and DMD modes, exactly as in the simulations (Fig. 6C and S15). Upon dimensionality reduction of the resulting 50 DMD amplitudes using kernelPCA again four populations were identified by k-means clustering (Fig. 6D). Almost 80% of the cell traces in cluster 1 are exposed to 30 mM glucose (cell 1-110; Fig. 6E). Cluster 2 and 4 consists to ~ 70% and 100% of cells exposed to 15 mM glucose, respectively (Fig. 6F and H), while only 10% of those cells belong to cluster 3 (Fig. 6G).

These results demonstrate that the concentration of added glucose is an important control point in determining metabolic oscillations in yeast, and that at intermediate concentrations (i.e. 15 mM glucose), oscillations are most pronounced in *hem1*Δ cells. These findings are in agreement with the simulations of our glycolysis model. That extracellular glucose concentration affects the extent of oscillations in yeast is also in accordance with other experimental studies^38,46^. Importantly, this analysis shows that combining DMD with TDE and machine-learning-based data clustering enables one to identify unique populations of metabolic oscillations in yeast cells.

### Yeast cells lacking Npc-proteins have altered metabolic Oscillation dynamics

Niemann Pick type C disease is a neurodegenerative disorder characterized by lack of either NPC1 or NPC2 protein, which leads to severe accumulation of cholesterol and sphingolipids in lysosomes^57^. *S. cerevisiae* expresses homologs of NPC1, named Ncr1, and of NPC2, named Npc2, in the vacuole (the fungal equivalent of the lysosome), and expressing these proteins in human cells can rescue the lipid storage phenotype^58,59^. Both proteins play an important role in lipid transport through the yeast vacuole, particularly under starvation conditions^60–62^. Apart from sterols, Ncr1 and Npc2 can bind various sphingolipids, which are also highly elevated in cells lacking Ncr1^63–65^. Accumulation of sphingolipids alters mitochondrial activity in various genetic backgrounds, including yeast lacking Ncr1, and it also results in impaired activity of the Snf1/AMPK protein kinase, a key regulator of energy homeostasis^64,66,67^.

**Fig. 7:**
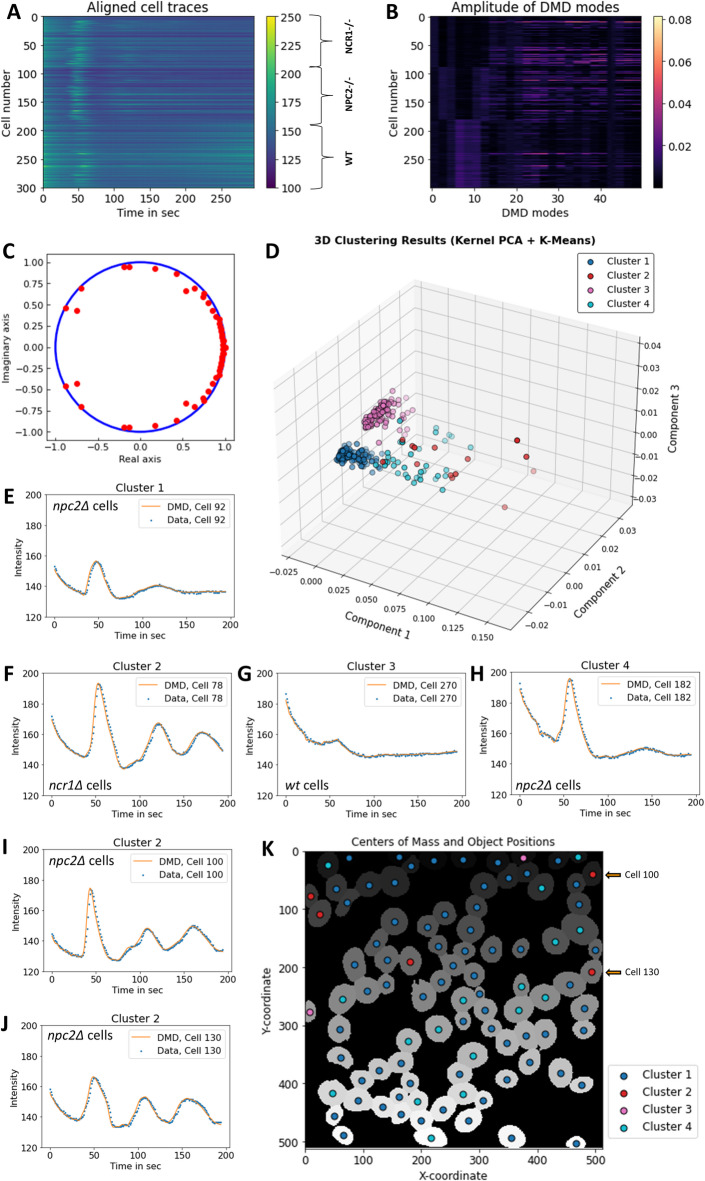
Classification of metabolic oscillations in yeast lacking Ncr1 or Npc2. The *wt*, *ncr1Δ* and *npc2Δ* cells were starved for 3 h in SD-medium without glucose before imaging. A bolus of glucose to a final concentration of 30 mM, was added after 30 s image recording. Cells were imaged for 300 s in total, intensity traces were extracted after segmentation and arranged as matrix concatenated for all conditions, (**A**) DMD with delay embedding, *d* = 100 and rank *r* = 50 was applied and mode amplitudes (**B**) and eigenvalues (**C**) were calculated. A kernel PCA was applied to the amplitude matrix, and the first three PCs were clustered into four clusters using k-means clustering, (**D**–**J**) shows example trajectories for each cluster (blue dots, data; orange line, DMD reconstruction). The cell mask obtained upon segmentation of *npc2Δ* cells is overlayed with the center of mass for each cell, color-coded according to affiliation into each group. Cluster 2 (red spheres) is characterized by three oscillation peaks in the interval of 0 to 200 s and was found predominantly in the knockout cells (see Tab. S1 and panel **F**, **I** and **J** for examples). The two oscillation profiles of panels I and J correspond to the two cells indicated by arrows in (**K**).

Since Snf1/AMPK also regulates the expression of glucose importers, we speculated that yeast lacking Ncr1 (*ncr1Δ* cells) or Npc2 (*npc2Δ* cells) might have altered NAD(P)H oscillations compared to wild-type (*wt* cells). To test this hypothesis, all three cell types were pre-starved before adding 3 mM glucose and recording NAD(P)H fluctuations, as described above. By applying DMD to the concatenated cell-time trace matrices and classifying cell trajectories based on the calculated mode amplitudes, we could indeed identify four clusters (Fig. 7A–D and Tab. S1). Cluster 1 only contained *ncr1Δ* and *npc2Δ* cells but no *wt* cells (Fig. 7D, blue spheres and Fig. 7E). More than 90% of cells in cluster 2 were either *ncr1Δ* or *npc2Δ* cells (Fig. 7D, red spheres). This population is characterized by three NAD(P)H spikes upon glucose addition (Fig. 7F, I and K).

Cluster 3 consisted of more than 98% of *wt* cells, the rest (i.e. 2 out of 111 cells) being *npc2Δ* cells (Fig. 7D, rosa spheres). This group shows only a transient upon glucose addition but no oscillations (Fig. 7G). Finally, cluster 4, which contains cells from all three cell types (see Tab. S1 for numbers), is characterized by an intense peak upon glucose addition, followed by a strongly damped and broad second peak (Fig. 7D, cyan spheres and Fig. 7H).

These results clearly show that DMD analysis of metabolic oscillations combined with machine-learning-based clustering of mode amplitudes can lead to the discovery of even subtle cellular phenotypes. Cells lacking either Ncr1 or Npc2 form distinct clusters in our classification analysis, with more pronounced oscillations compared to control cells, indicating that both proteins play a central role in overall energy homeostasis.

Since the temporal profiles of cellular autofluorescence were extracted from segmented time-lapse videos, we can map the coordinates of each cell’s center of mass together with the cluster identity back to the cell images (Fig. 7J). This enables us to identify cells of a given cluster (i.e. with a cluster-specific oscillation signature) in the video data (Fig. 7J, arrows pointing to cells whose intensity profile is shown in Fig. 7I and K for *npc2Δ* cells). This feature is very important, as it opens up for many additional single-cell studies in the future. An example is the analysis of cell-to-cell communication during metabolic oscillations leading to population-density-dependent oscillations^68,69^. Our single-cell analysis pipeline can also be combined with co-staining of organelles for advanced cell phenotyping.

## Discussion

Metabolic oscillations are a widespread phenomenon and occur not only in glycolysis of fermenting yeast cells but also in glycolysis of cancer cells, in pancreatic β-cells in connection with pulsatile insulin secretion, as well as in muscle cells and human neutrophils^1,35^. A lot of theoretical work has been dedicated towards obtaining a mechanistic understanding of such oscillations, but little focus has been directed towards employing modern data-driven methods for quantitative analysis of such oscillations based on time-lapse microscopy of individual cells^13,16^.

Here, we show that DMD combined with TDE allows for faithful reconstruction and modal decomposition of simulated and experimental glycolytic oscillations. We show that the eigenvalue spectrum determined by DMD for simulated and experimental metabolic oscillations depends on the delay embedding dimension. For increasing delays, the spectrum converges and becomes stable with low residuals, showing that under those conditions, DMD recovers a good linear approximation of the Koopman operator, capturing essential elements of the system dynamics. The embedding dimension plays a central role in approximating the dynamic manifold for the oscillations, which is a result of Taken’s theorem^26^. We demonstrate that this is even the case for non-stationary dynamics and in the presence of noise, as long as the system is relatively close to a stable steady state.

However, we also find that DMD’s ability to predict future time points is limited for both simulated and experimental glycolytic oscillations. The limited performance in predicting future time points is a direct consequence of the assumptions underlying any DMD analysis, namely that the dynamics are approximately linear. More precisely, DMD assumes that a linear subspace of the Koopman operator exists, which captures the essential system dynamics as a linear superposition of exponential eigenfunctions with constant amplitudes and eigenvalues. The validity of this assumption, however, is questionable in situations where the system quickly moves away from the fixed point, i.e., in which the Jacobian defining the tangent space poorly describes the system dynamics (see Appendix)^49^. In these situations, higher-order terms of the Taylor expansion around the steady state play an important role, and the linearity assumption becomes questionable, particularly in the presence of noise. While inclusion of higher-order terms, as in HoDMD as well as TDE, increases the state space, these DMD variants are still linear methods, as they attempt to find a finite-dimensional approximation to the (linear) Koopman operator. Thus, the DMD approach does not explicitly model the non-linear terms that characterize the system’s true behavior. Instead, the inclusion of higher derivatives, as in HoDMD, or the use of time-shifted versions of the data, as in DMD with TDE, leverages additional information about the system to refine the linear approximation.

By combining DMD with TDE and data clustering, we show that mode amplitudes can be used for classification of single-cell trajectories. This is an important application of DMD, which allows us to identify subtle changes in oscillatory behaviour of single cells upon altering external glucose concentration. Thus, we show in both the simulations and the experiments that glucose influx is an important control parameter of glycolytic oscillations, as was previously suggested based on bulk cuvette experiments combined with kinetic modelling^46,47,70^.

Single-cell analysis provides additional insight compared to such earlier studies, as it accounts for cell-to-cell heterogeneity and does not rely on synchronous behavior of the entire cell population^38,56^. This is important, since even small changes in frequency between individual cells can lead to dephasing of the population, which would result in broadening and rapid decay of the oscillatory behavior in cuvette experiments^13,16,33,68^. Analyzing cell-to-cell variability in oscillations between genetically identical cells, which are cultured under identical conditions, offers additional insight into the stochastic nature of the underlying metabolic networks. For example, the observed cell-to-cell heterogeneity in glycolytic oscillations for varying external glucose concentrations could be due to differences in molecular abundance of hexose transporters between individual cells^48^. Their expression is controlled by glucose, which is sensed by the Snf3/Rtg2 or Gpr1/Gpa2 systems, resulting in expression of primarily low-affinity transporters Hxt1 and Hxt3 at high external glucose and high-affinity Hxt2, 4, 6 and 7 at low glucose concentrations^71^. For isogenic cells cultured under identical conditions, the expression level of fluorescence-tagged Hxt7 has been determined and found to vary 10-fold between individual cells^48^. Similar cell-to-cell variation likely occurs for other glycolysis enzymes, and that we can pick up even small changes in metabolic activity as a consequence of altered enzyme abundance shows the strength of our analysis method. Note that the *hem1Δ* cells have defective oxidative phosphorylation, such that these cells only rely on glycolysis and fermentation, but not on respiration.

Using DMD with TDE on autofluorescence fluctuations in *ncr1Δ* and *ncr2Δ* cells, we found that yeast Ncr1 and Npc2, which are known for their role in lipid transport and vacuole fusion^58–60,62^, also play a role in metabolic oscillations. We observe enhanced oscillations in a subpopulation of yeast lacking either Ncr1 or Npc2 compared to wild-type cells. For these strains the background is BY4741/2, which is able to respirate. At this point, we can only speculate why *ncr1Δ* and *npc2Δ* cells have more pronounced oscillations than *wt* cells, but one attractive hypothesis is that defective lipid transport impacts the energy homeostasis of the cells. For example, build-up of sphingolipids has been shown to be accompanied by dysfunction of mitochondria in various genetic backgrounds, including *ncr1Δ* cells^64,67^. Supporting that notion, we found in preliminary proteomics experiments that the same *ncr1Δ* and *ncr2Δ* cells show altered expression of enzymes in the sphingolipid synthesis pathway but also in glycolytic and respiratory enzymes compared to control cells (Thaysen et al., manuscript in preparation). Such changes could cause altered metabolic oscillations, which we can detect by our new method.

Using TDE as the coordinate basis for data-driven representation of the Koopman operator is powerful, as shown here for glycolytic oscillations. But our method can also be used to study other types of biochemical oscillations. In fact, we found in additional experiments that DMD with TDE also reconstructs simulated calcium oscillations very well, much better than the traditionally used Fourier decomposition (Fig. S16). Thus, applying DMD with TDE bears large potential for many other applications, including analysis of oscillating signals in neuroscience experiments^41,72^ or control of the cell cycle^73^. To further improve the performance of this approach, one could explore non-uniform delay embedding strategies, as reviewed and suggested in^50^. One could also explore alternative observables, for example, using a dictionary of non-linear functions of the snapshot data as observables, as suggested in various forms of extended DMD^42,74^. By using such observables, one would eventually increase the likelihood of finding good eigenfunctions of the Koopman operator, which adequately describe the entire manifold. However, finding such observables is not trivial, and a lot of current efforts are dedicated to this goal^75,76^. One possibility is to combine DMD with neural-network-based approaches in the future. This would aim to learn a faithful linear encoding of the non-linear dynamics, allowing for improved finite-dimensional approximation of the Koopman operator^74,76–83^.

Alternatively, the DMD approach could be informed about forcing terms, which exert control of the studied dynamic system. This approach is also widely used, and when combined with delay embedding, it was found to describe even chaotic systems^29,84^. Finally, the assumption of eigenfunctions being constant over the entire time series can be replaced by dynamic modes varying along the studied dynamics. This has been implemented as multiscale reconstruction, similar to wavelet analysis^18^, and as a windowed approach in the recently developed non-stationary DMD^85^. Like some of the autoencoder-based DMD variants^81^ these approaches might be better suited to analyze non-stationary oscillations, and they will be explored for imaging-based analysis of the dynamic metabolism of living cells in the future.

## Materials and methods

### The standard DMD algorithm applied to simulated and experimental time courses

The theory behind DMD and HoDMD is described in^25,86^, and follows the procedure outlined in our recent publications^23,24,87^. Briefly, snapshots in time (i.e., concentration of metabolite species or fluorescence intensities of NADH, either as single measurements or as images) are arranged in a sequence^22,88^:

1 $$\:{X}_{1}=\left[\stackrel{-}{{x}_{1}},\:\stackrel{-}{{x}_{2}},\:\cdots\:,\stackrel{-}{{x}_{m-1}}\right]$$.

and

2 $$\:{X}_{2}=\left[\stackrel{-}{{x}_{2}},\:\stackrel{-}{{x}_{3}},\:\cdots\:,\stackrel{-}{{x}_{m}}\right]$$.

The index *k* = 1,…, *m* runs over all acquired snapshots in time with steps, Δt. One can define the progression from state x(k·Δt) = x_k_ to x_k+1_ as:

3 $$\:{x}_{k+1}=A \cdot \:{x}_{k}\:$$.

Here, *A* is a matrix which describes the advancement of the system from image *x*_k_ to image *x*_k+1_. This matrix resembles the Koopman or transfer operator for measurements g(*x*_k_) = *x*_k_^86^. With that the system corresponding to Eq. (3) becomes *X*_2_ = *A*·*X*_*1*_, from which we can find *A* by minimizing the Frobenius norm, $\:{\vert\vert \cdot \:\vert\vert}_{F}$^86^:

4 $$\:A:=argmin{\vert\vert{X}_{2}-A \cdot \:{X}_{1}\vert\vert}_{F}={X}_{2} \cdot \:{X}_{1}^{inv}$$.

To solve this system, one first finds the pseudoinverse of the first data matrix, $\:{X}_{1}^{inv}$. For that, one uses a SVD of *X*_1_ into unitary matrices U $\:\in\:$ *R*^n·(m−1)^, V*$\:\in\:$ *R*^n·n^ with singular values in the diagonal matrix Σ $\:\in\:$ *R*^n·(m−1)^:

5 $${X_1}=U \cdot \sum \cdot V*$$.

To obtain a low-rank approximation of the dynamic only the modes up to rank *r* < (*m*-1) are retained, i.e., one approximates the system matrix *A* by projecting it onto the left singular vectors, which are the column vectors of U. This gives a much smaller matrix A’ of maximal size *m* times *m* via a similarity transformation^20,86^:

6 $$A^{\prime}=U^{\prime}* \cdot A \cdot U^{\prime}=U^{\prime}* \cdot {X_2} \cdot V^{\prime} \cdot {S{^{\prime-1}}}$$.

Here, *U*’, *V*’ and *Σ*’ are rank *r* approximations of the full matrices, *U*, *V* and *Σ*, and the complex conjugate transposed matrix is indicated by *:

7 $$A^{\prime}={U^{\prime T}} \cdot A \cdot U^{\prime}={U{^{\prime T}} \cdot {X_2} \cdot V^{\prime} \cdot {S^{\prime-1}}}$$.

The similarity transformation of Eq. (6) corresponds to a dimension reduction, which reduces the size of the system matrix from *A* $\:\in\:$ *R*^(m−1)∙(m−1)^ to *A’*$\:\in\:$ *R*^r∙r^ giving a low-dimensional representation of the dynamic system^86^. To obtain the spectral decomposition of the reduced system, one finds the eigenvalues, *λ*_*j*_ and eigenfunctions, *φ*_*j*_, for each DMD mode *j*, by solving the corresponding eigenvalue problem:

8 $$\:{x}_{k}=\sum\:_{j=1}^{r}{\phi\:}_{j} \cdot {\lambda\:}_{j}^{k-1} \cdot {b}_{j}$$.

For a continuous system, e.g. in time, one can rescale the eigenvalues according to *ω* = ln(*λ*/Δ*t*), such that Eq. (8) can be written as^19^:

9 $$\:x\left(t\right)=\sum\:_{j=1}^{r}{\phi\:}_{j} \cdot {e}^{{\omega\:}_{j} \cdot \:t} \cdot {b}_{j}$$.

Here, *x*(*t*) is a vector of images (x, y index omitted for brevity) as a function of time, *t*,

### Extension of the DMD algorithm to include time-shifted versions of the snapshot data

The matrix *A* can be approximated from the data by first introducing the higher-order Koopman assumption, which reads

10 $$\:{x}_{k+d}={A}_{1} \cdot \:{x}_{k}+{A}_{2} \cdot \:{x}_{k+1}+..+{A}_{d} \cdot \:{x}_{k+d-1}$$.

Here, *k* runs from 1 to *m*-*d*, where *m* is the number of snapshots taken at discrete time steps and *d* is the set time-delay. A rank-truncated SVD, as described in Eq. 5 to 7 is applied to the snapshot matrix leading to:

11 $$\:{\stackrel{\sim}{X}}_{k+1}=A{\prime\:} \cdot \:{\stackrel{\sim}{X}}_{k}$$.

The reduced matrices for the snapshots and the evolution of the system read^25^:

12 $$\:{\stackrel{\sim}{X}}_{k}=\left(\begin{array}{c}{x}_{k}\\\:{x}_{k+1}\\\:\dots\:\\\:{x}_{k+d-1}\end{array}\right)\:,\:A{\prime\:}=\left(\begin{array}{cccc}I&\:0&\:\dots\:&\:0\\\:0&\:I&\:\dots\:&\:0\\\:\dots\:&\:\dots\:&\:\dots\:&\:0\\\:{A}_{1}&\:{A}_{2}&\:\dots\:&\:{A}_{d}\end{array}\right)$$.

HoDMD uses this approach and was employed on the simulated and image data as implemented in the PyDMD package^40^. An alternative approach for time-delay embedding is to construct a Hankel matrix of the data matrix of Eq. (1) by including time-shifted versions of each row according to:

13 $$\:H=\left(\begin{array}{cccc}{x}_{1}\left({t}_{1}\right)&\:{x}_{1}\left({t}_{2}\right)&\:\dots\:&\:{x}_{1}\left({t}_{n}\right)\\\:{x}_{2}\left({t}_{1}\right)&\:{x}_{2}\left({t}_{2}\right)&\:\dots\:&\:{x}_{2}\left({t}_{n}\right)\\\:\dots\:&\:\dots\:&\:\dots\:&\:\dots\:\\\:{x}_{m}\left({t}_{1}\right)&\:{x}_{m}\left({t}_{2}\right)&\:\dots\:&\:{x}_{m}\left({t}_{n}\right)\\\:{x}_{1}({t}_{1}+l)&\:{x}_{1}({t}_{2}+l)&\:\dots\:&\:1({t}_{n}+l)\\\:\dots\:&\:\dots\:&\:\dots\:&\:\dots\:\\\:{x}_{m}({t}_{1}+d)&\:{x}_{m}({t}_{2}+d)&\:\dots\:&\:{x}_{m}({t}_{n}+d)\end{array}\right)$$.

Here, *t*_i_, *i* = 1, …*n* are the snapshots and *x*_j_ = 1, …*m* are the individual time traces, i.e., the simulated or experimental time courses for each cell. The index *l* is the time delay (chosen as *l* = 1 or 5) and *d* is the embedding dimension, i.e., the total number of chosen time shifts. The matrix H and its time-advanced variant were used in the standard DMD algorithm according to Eq. 1 to 9, above.

### Yeast cell culture, live-cell imaging and cell segmentation

The cells were grown in YPD media consisting of 2% (w/v) D(+)-Glucose Monohydrate (Merck, 1.08342.1000), 2% (w/v) Bacto Peptone (BD Chemicals, 211,677), 1% (w/v) Yeast Extract (Merck, 1.03753.0500) and 0.02% (w/v) adenine (Sigma-Aldrich, A-2786). The yeast strain *hem1*Δ is derived from the W303-1*α* strain, and it was kindly made available by Dr. Thomas Pomorski (University of Copenhagen, Section Transport Biology). *hem1*Δ cells were grown in YPD media with 50 µg/ml δ-aminolevulinic acid (dALA) (Sigma-Aldrich, A3785). As wild-type for the experiments on Ncr1- and Npc2-deficient cells, we used a BY4741-strain (purchased from Euroscarf), and *ncr1Δ* and *npc2Δ* in the BY4742 background were kindly provided by Prof. Christopher Beh (Simon Fraser University, Department of Molecular Biology and Biochemistry).

Before imaging, the media was changed to SD media without glucose consisting of 0.001% (w/v) D(+)-Glucose Monohydrate (Merck, 1.08342.1000), 0.7% (w/v) Yeast Nitrogen Base w/o amino acids (BD Difco, 291920), 0.2% (w/v) Yeast Synthetic Drop-Out Medium Supplements without uracil (Sigma-Aldrich, Y1501), 0.012% (w/v) adenine (Sigma-Aldrich, A-2786) and 0.002% (w/v) uracil (Sigma-Aldrich, U1128) and incubated in a shaking incubator (~ 30 °C, 150 rpm) for 3 h to ensure glucose depletion. The yeast was diluted to 2 OD_600_/ml in PBS before the cells were added to a microscope dish. During imaging, glucose was added to the dish to a final concentration of 3, 15 or 30 mM. Imaging was conducted on a Leica DMIRBE fluorescence microscope with a 100 × 1.3 NA oil immersion objective with a sampling rate of 1 Hz. The NAD(P)H autofluorescence was obtained using an A4 filter cube with a 360 nm (40 nm bandpass) excitation filter, 400 nm dichromatic mirror and a 470 nm (40 nm bandpass) emission filter.

Each cell was segmented based on the max Z-projection of the fluorescence image using the built-in Cyto2 model with a diameter of 40 in the deep learning-based segmentation method, CellPose, as described^89^.

### Model simulations and parameter assessment

Simulations of the glycolysis and the calcium model were implemented in Python and carried out either at the single-cell or the population level, as described in the Results section. In most simulations, multiplicative noise drawn from a log-normal distribution was added to mimic stochastic fluctuations stemming from moderately low substrate concentration or enzyme copy numbers working under non-saturating conditions with feedback loop. The calcium model was originally described for astrocytes and is described in^90^.

The LSTM analysis was conducted on the averaged experimental data. The LSTM model was implemented using PyTorch, with mean squared error as the loss function and the Adam algorithm as the optimizer. Once the model was set up, the parameters to be tested were defined, including the number of layers and nodes in the network, as well as the proportion of data used for training. After defining these parameters, the training process was initiated. The network was trained until the loss function no longer decreased, typically after 100–200 epochs. For analysis, the model’s predicted data was plotted against the real, unseen experimental data, and a quantitative evaluation was performed by calculating the R² value.

The accuracy of the DMD reconstruction was assessed using the L_2_-error, the Pearson coefficient and the R^2^-score. While the L_2_-error measures the distance between simulated and reconstructed time series in a point-wise manner, the Pearson coefficient quantifies, how much they correlate (go up and down together; *r* = 1 is perfect correlation). The R^2^-score quantifies how much variance in the data is explained by the model.

DMD amplitudes were calculated as square root of the sum of the real and imaginary parts of the eigenfunctions, and their classification was achieved by first applying kernel PCA followed by k-Means clustering of the first three PCs. The choice of cluster number was based on the Sillouette criterion, and all calculations used the Python library scikit-learn^91^.

## Electronic supplementary material

Below is the link to the electronic supplementary material.

Supplementary Material 1

Supplementary Material 2

## Data Availability

The datasets used and analysed during the current study are available from the corresponding author on reasonable request.
