# Supplementary material for: Dynamic mode decomposition for analysis and prediction of metabolic oscillations from time-lapse imaging of cellular autofluorescence
Source: Sci Rep. 2025 Jul 2;15:23489. doi: 10.1038/s41598-025-07255-4 (PMC12222966; doi:10.1038/s41598-025-07255-4)
Supplement: Supplementary file 1 — Supplementary Material 1 [file 41598_2025_7255_MOESM1_ESM.docx]

**Supplementary information for**

**Dynamic mode decomposition for analysis and prediction of metabolic oscillations from time-lapse imaging of cellular autofluorescence**

Daniel Wüstner^#^, Henrik Helge Gundestrup, Katja Thaysen,

Department of Biochemistry and Molecular Biology University of Southern Denmark, DK-5230 Odense M, Denmark

^#^Address correspondence to: Daniel Wüstner, Department of Biochemistry and Molecular Biology, University of Southern Denmark, Campusvej 55, DK-5230 Odense M, Denmark

Tel. +45-6550-2405, e-mail: wuestner@bmb.sdu.dk

**Metabolic oscillations in a minimal model of glycolysis are inferred by HoDMD**

Kinetic models of glycolysis in eukaryotic cells are primarily based on ordinary differential equation (ODE) systems, which can become rather complex depending on the purpose of the analysis [1-4]. Our model is based on the following system of non-linear ordinary differential equations:

$\frac{dS_{1}}{dt}=v-$k_1_∙S_1_+ k_1_∙S_1_∙(S_2_/K)^n^ (S1)

$\frac{dS_{2}}{dt}=$k_1_∙S_1_+ k_1_∙S_1_∙(S_2_/K)^n^- k_2_∙S_2_ (S2)

Here, S_1_ and S_2_ are the concentrations of fructose-6 phosphate (F6P) and fructose-1-6-bisphosphate (F16BP), respectively. The conversion of F6P into F16BP is described by rate constant *k*_1_, and the allosteric activation of PFK in this reaction is modeled by the Hill coefficient *n*, describing the extent of cooperativity of allosteric activation of PFK by F6P. The equilibrium constant for this binding step is *K*, and the rate constant for the aldolase reaction removing F16BP is *k*_2_. In our model, the positive feedback due to allosteric activation of PFK by F16BP will cause a continuous increase in production of S_2_ (i.e., F16BP), until S_1_ (i.e., F6P) is depleted. This causes a decline in the concentration of F16BP until more F6P due to conversion from G6P is available, and the cycle can begin again. If the aldolase step is slower, i.e. *k*_2_ =3 min^-1^, only damped oscillations are found, independent of the cooperativity of the feedback step, since this step separates regions of stable and unstable steady state (see Fig. S1 and [5]).

Using HoDMD, this data can be reconstructed almost perfectly (Fig. S3). More specifically, we find that the reconstructed time courses obtained with a delay constant of *d*=300 exactly coincide with the simulated ones, both in cases of damped and sustained oscillations. Glucose influx, *v*, and feedback cooperativity, *n*, control the type of oscillations occurring, and HoDMD can reconstruct both types of oscillations accurately. The DMD eigenvalues for the reconstructions shown in Fig. S1C-F are plotted on the unit circle in Fig. S2A-D. The denoising capacity of HoDMD is compared to that of the Fourier transform as illustrated in Fig. S2E-G. For that, the power spectral density was calculated using the Welch method as implemented in Scipy in Python and truncated for large frequencies representing noise for both F6P and F16BP (Fig. S2E and F). Denoising by HoDMD is based on rank-truncation of the singular value decomposition (SVD) and performs much better than denoising with the FFT (Fig. S2G).

| 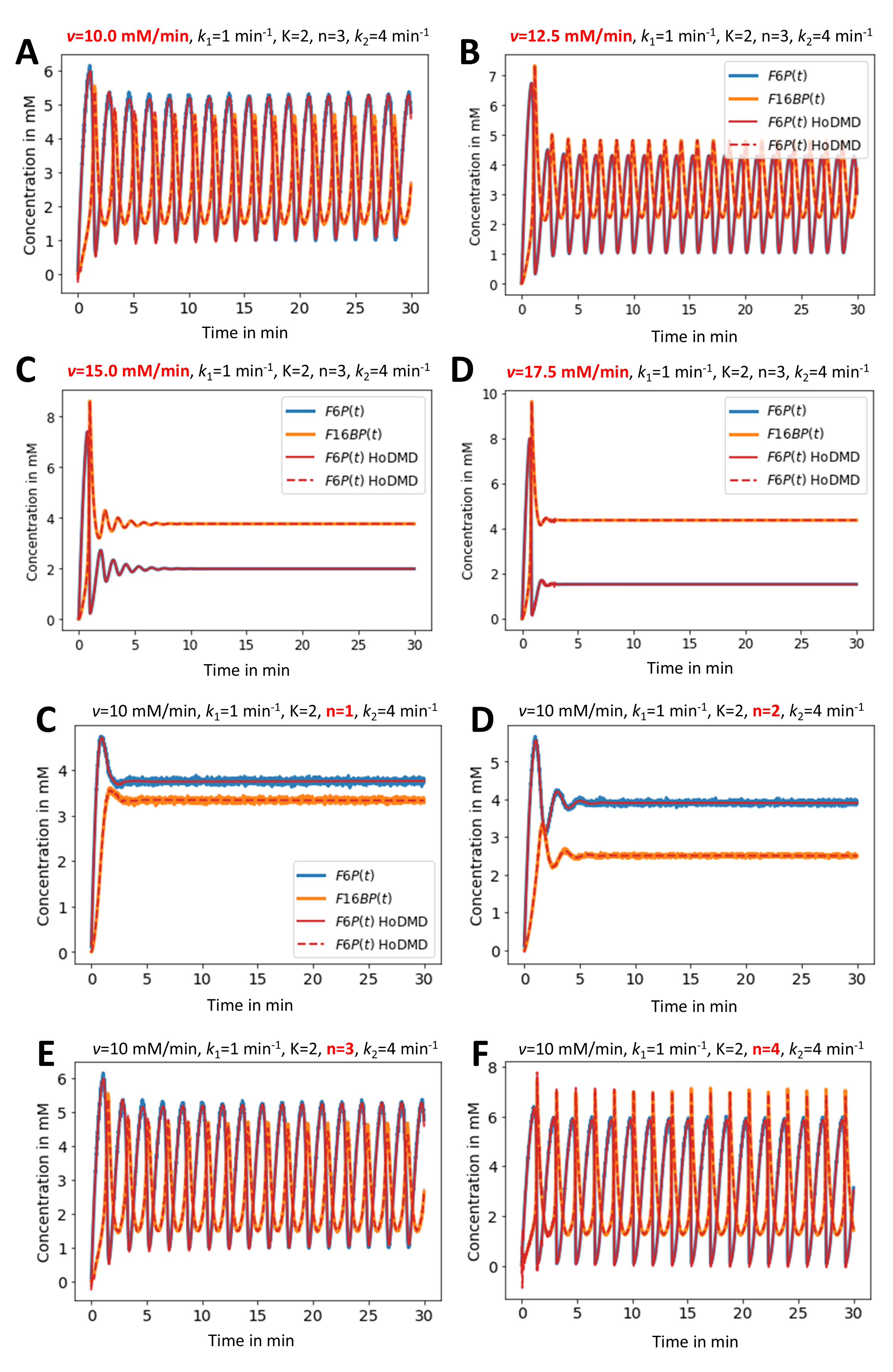 | **Figure S1. HoDMD**  **of the glycolysis model.** Time courses for F6P and F16BP were simulated with the minimal glycolysis model for varying glucose influx described by the rate v (red values on top of panels A-D, blue and orange dots show simulation of F6P and F16BP, respectively, while red straight and dashed lines show the corresponding HoDMD reconstructions). (C-F) simulated time courses for F6P (blue) and F16BP (orange) and their reconstruction by HoDMD in straight and dashed red lines, respectively, are shown for n=1 (C), n=2, (D), n=3 (E) and n=4 (F). All other parameters are as indicated on top of panel C-F. |
| --- | --- |

| **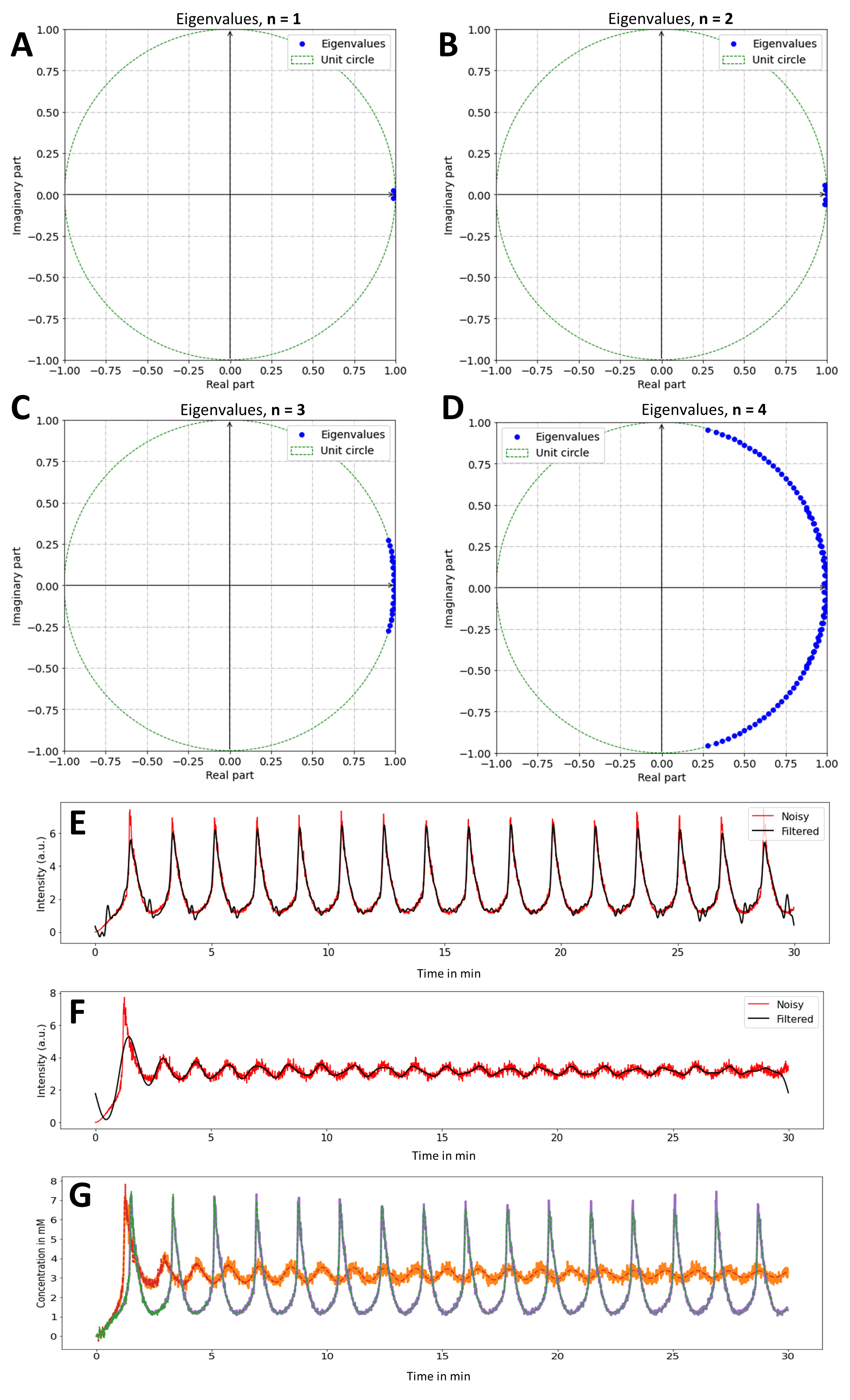** | **Figure S2. Eigenvalues of HoDMD analysis and comparison with Fourier filtering.** The eigenvalue spectrum of the HoDMD analysis of the simulated time courses for varying Hill coefficients, *n*, is shown in A-D. E and F show simulated time courses for F6P (E) and F16BP (F) with noise (red lines) and the resulting Fourier reconstruction (black lines). The reconstruction of the same data by HoDMD with rank truncation of r= 20 is shown in panel G for F6P (violet line, data; green line, reconstruction) and for F16BP (orange line, data; red line, reconstruction). See text for further details. | |
| --- | --- | --- |
|  | |  |
|  | |  |
| **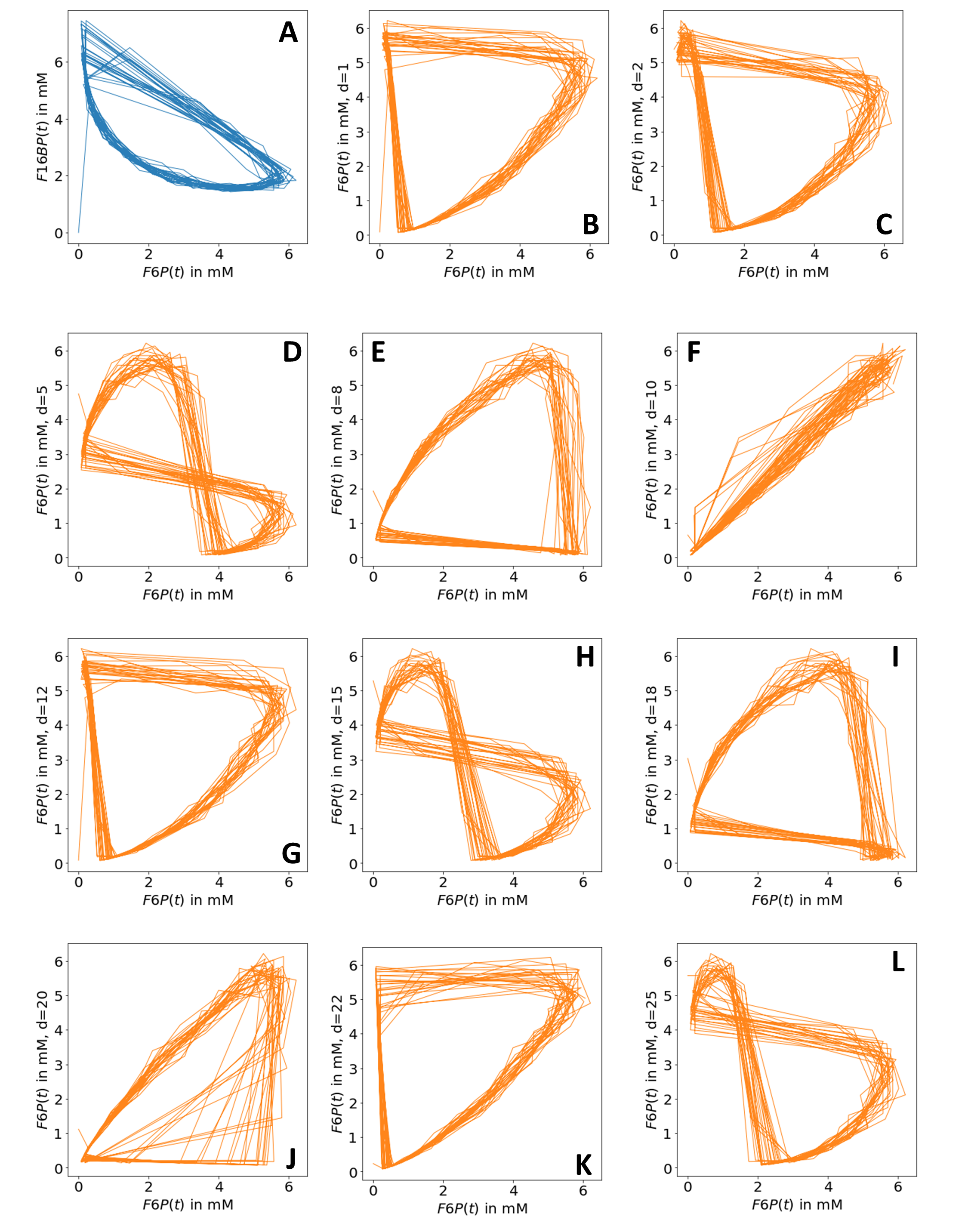** | |  |
| **Figure S3. Time-delay embedding can approximate the attractor of the glycolysis model.** The glycolysis model was simulated for the parameters v=9.5 mM/min, k_1_=1 s^-1^, K=2, n=4 and k_2_=4 s^-1^ with added log-normal noise, as described in Fig. 1 and 3 of the main text. A, phase plot of time course for F16BP plotted against that of F6P. B-L, time course of F6P plotted as function of time-shifted versions of itself for increasing time-shift (delay, *d*); i.e., for *d*=1 (B), 2 (C), 5 (D), 8 (E), 10 (F), 12 (G), 15 (H), 18 (I), 20 (J), 22 (K) and 25 (L). For certain values of *d*, the shadow manifold M' is diffeomorphic, i.e., a 1:1 embedding of the original manifold (here for *d*=1, 12, 20 and 22, while for others it is not, due to crossings (here for *d*=2, 5, 15, 18 and 25) or collapse of the manifold (here for *d*=10). Note the similarity of the phase plots for every 10 steps of *d* (e.g. in panel D, H and L or in panel E vs. I and in panel C, G and K). | |  |

**Stability analysis of the two-state glycolysis model**

By setting Eqs. S1 and S2 to zero, one finds expressions for the steady state concentration of F6P and F16BP (see Appendix, Eqs. A1 and A2). The stability of the dynamic system can be assessed by a Taylor expansion around this steady state and truncating the series after the first term leading to the Jacobian matrix of the system [5, 6]. The Jacobian of this simplified glucose model was solved analytically and is given in Eqs. A3 to A7 of the Appendix. The eigenvalues of this matrix are derived in Eqs. A8 and A9. They are plotted for different values of *k*_2_, the rate constant for the aldolase reaction, as a function of glucose influx for a Hill coefficient of n=3 in Fig. S1A-D. For *k*_2_ =3 min^-1^, and either low (*v*_0_ < 7.5 mM/min) or high (*v*_0_ > 12.5 mM/min) glucose influx the eigenvalues have both negative real parts, showing that the steady state is stable for these cases. Thus, after a small perturbation, the dynamics of the system will asymptotically return to the steady state for these parameter values. In contrast, for intermediate glucose concentrations, the system is unstable, as inferred from the eigenvalues having positive parts (Fig. S4A and C). In general, a steady state of such a two-component system is stable as long as the trace of the Jacobian is negative, while its determinant is positive [5, 6]. One can see from Fig. S4E-G and Eq. A10, that the determinant of the Jacobian is positive for all parameter combinations, while its trace changes sign for intermediate glucose influx, which is more pronounced for *k*_2_=4 min^-1^ compared to *k*_2_=3 min^-1^. This change of sign of the trace of the Jacobian is characteristic for a Hopf bifurcation, which happens at the bifurcation point of n=2.4 for this system [5, 6]. For positive trace of the Jacobian, which happens for intermediate glucose influx, high cooperativity of the feedback and fast aldolase reaction (i.e., *k*_2_=4 min^-1^), the steady state becomes unstable, and from the plot of trace squared minus four times the determinant, one sees that an unstable spiral forms under those conditions (Fig. S4E-G). This is the region, in which the system establishes a limit cycle, i.e. sustained oscillations in the abundance of both metabolites. In contrast, for *k*_2_=3 min^-1^ and glucose ≥ 10 mM/min, a stable focus (also called a stable spiral) forms with damped oscillations (Fig. S4D-F). For high glucose influx (i.e., *v* ≥ 17 mM/min), the stable focus is found for fast aldolase reaction (i.e., *k*_2_=4 min^-1^), but the dynamic behavior changes to a stable node for slower aldolase reaction (i.e., *k*_2_=3 min^-1^, Fig. S4D-F). The latter is characterized by the absence of oscillations. The limit cycle observed for n=3 and n=4 is in accordance with the Poincare-Bendixson theorem, which states, that as long as the trajectories of the species are bounded on the manifold M for t → ∞, a closed cycle is formed [5, 6]. The corresponding eigenvalues reflect the change in system behavior for increasing feedback cooperativity and glucose influx (Fig. S4A-D).

| 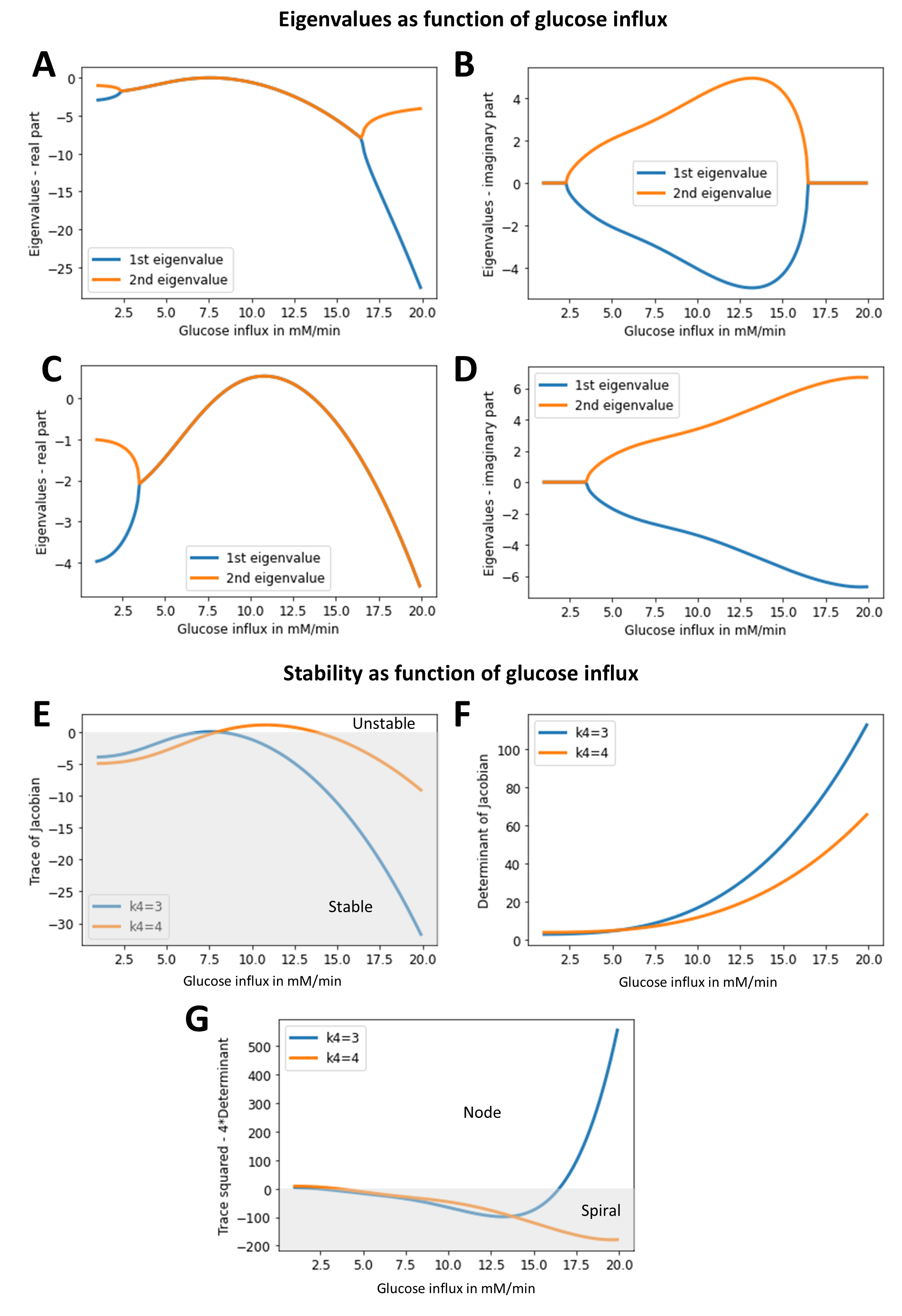 | **Figure S4. Stability of the steady state determined for the minimal glycolytic model.**  Based on the calculated steady state values for F6P and F16BP, the eigenvalues of the system were calculated for *k*_2_= 3 min^-1^ (A and B) and *k*_2_= 4 min^-1^ (C and D), and the real part (A and C) as well as imaginary part (B and D) of these eigenvalues are plotted as function of glucose influx, *v*. E-G, a stability analysis was performed as function of glucose influx, showing the trace (E) and determinant of the Jacobian (F) for the rate constant of the aldolase reaction set to *k*_2_=3 min^-1^ (blue lines) or to *k*_2_=4 min^-1^ (orange lines). |
| --- | --- |
| The sign of the difference between the trace of the Jacobian and four times its determinant informs about the nature of the observed steady state (G). See text and Appendix for further explanations. | |

| **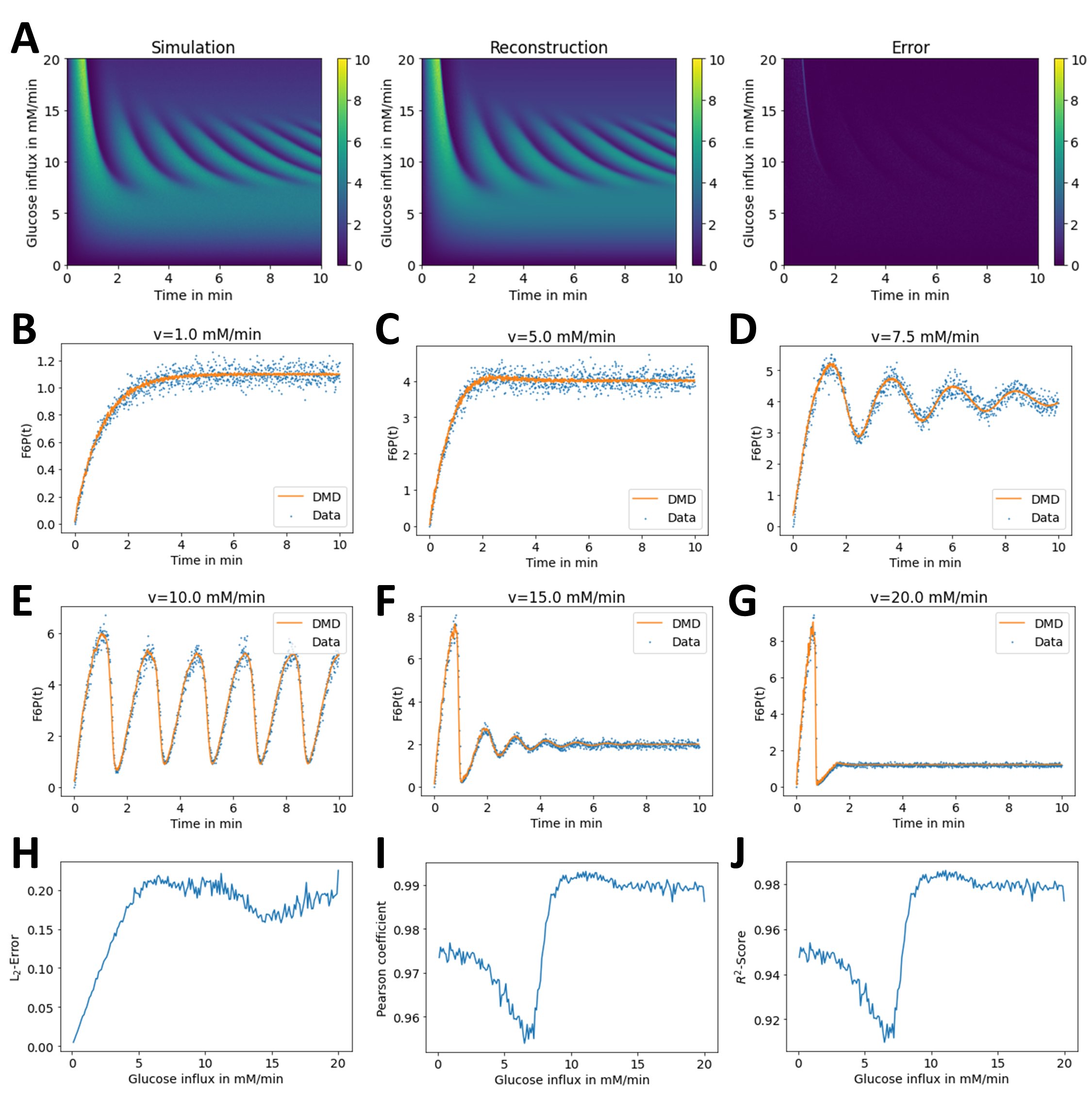** |
| --- |
| **Figure S5. Assessment of reconstruction performance of DMD with TDE for systematically varied glucose influx.** The glycolysis model was simulated with the parameters *k*_1_ = 1 min^-1^, *k*_2_, = 4 min^-1^, K= 2 and *n*=3, while the glucose influx, *v*, was varied between 0.1 mM/min and 20 mM/min in steps of Δ*v* = 0.1 mM/min. Noise was added from a log-normal distribution around the mean with σ=0.015. For each of the resulting 200 trajectories a TDE scheme was implemented with delay, *d*=500. Each of the resulting 200 Hankel matrices was analyzed by DMD with rank, r=50. A, the simulated time courses (left panel), the DMD reconstruction (middle panel) and the L_2_-error (right panel). B-G, selected trajectories for varying glucose influx (blue dots, data; orange line, DMD reconstruction). The corresponding influx value for each simulation is given on top of each panel. H-J, various measures of error between simulated and reconstructed data, including the L_2_-error (H), the Pearson coefficient (I) and the R^2^-score (J). All three measures show that the reconstruction of the simulated data is very good over the entire parameter range. |

| **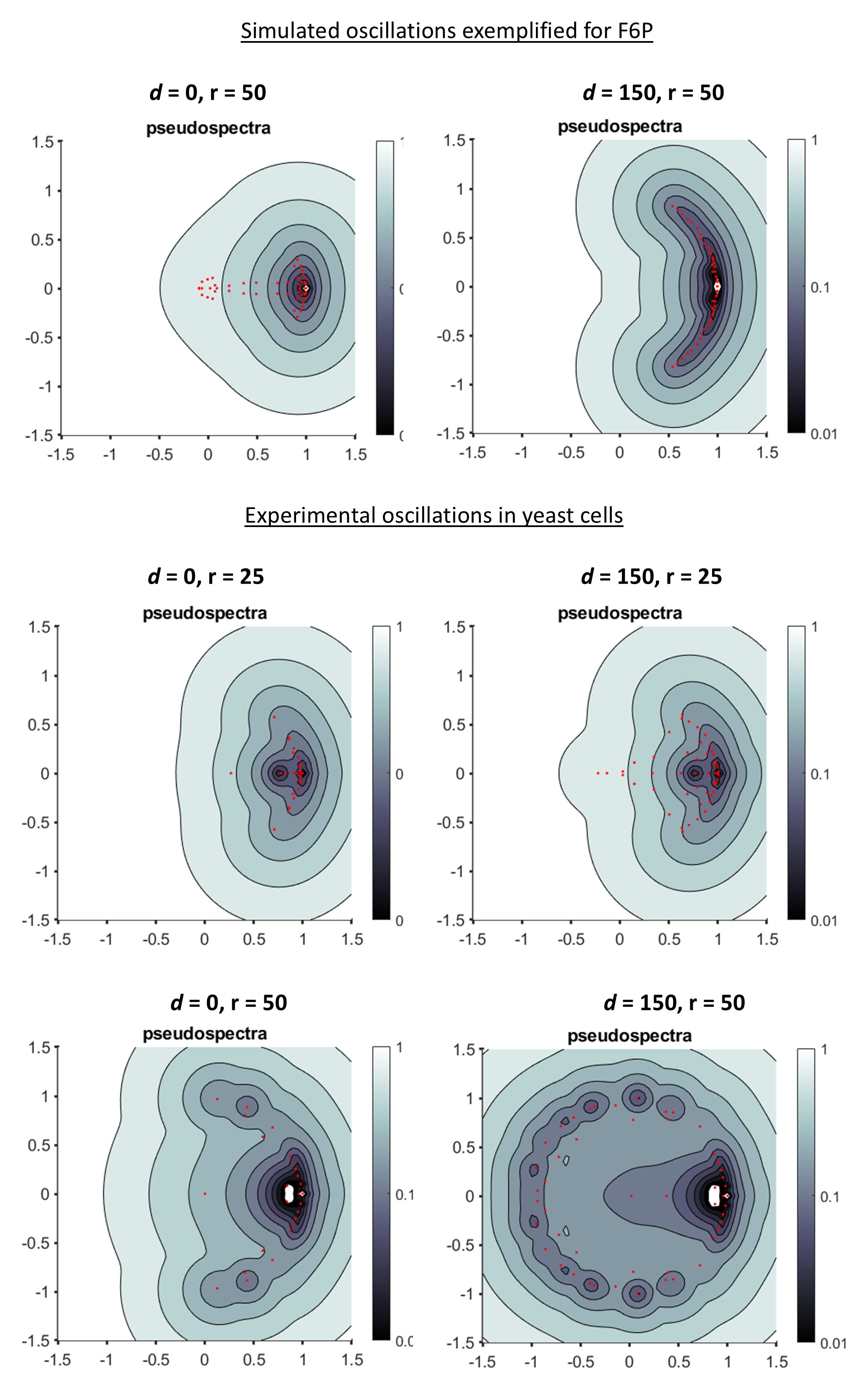** |
| --- |
| **Figure S6. Eigenvalue spectrum of synthetic and experimental oscillations by residual DMD.** Residual DMD was applied to cell-time matrices of the simulated data (upper panel) and of the experimental data (lower panel), either on the original data matrices without delay (*d* = 0) or on the Hankel matrices (*d* = 150) with rank truncation of r = 25 and r = 25 or 50 for the synthetic and experimental data, respectively. Red dots show the identified eigenvalues on the unit circle, and grey shades are the associated residuals with dark grey indicating lower residuals and thereby higher confidence in the determined eigenvalues compared to light grey. |

| **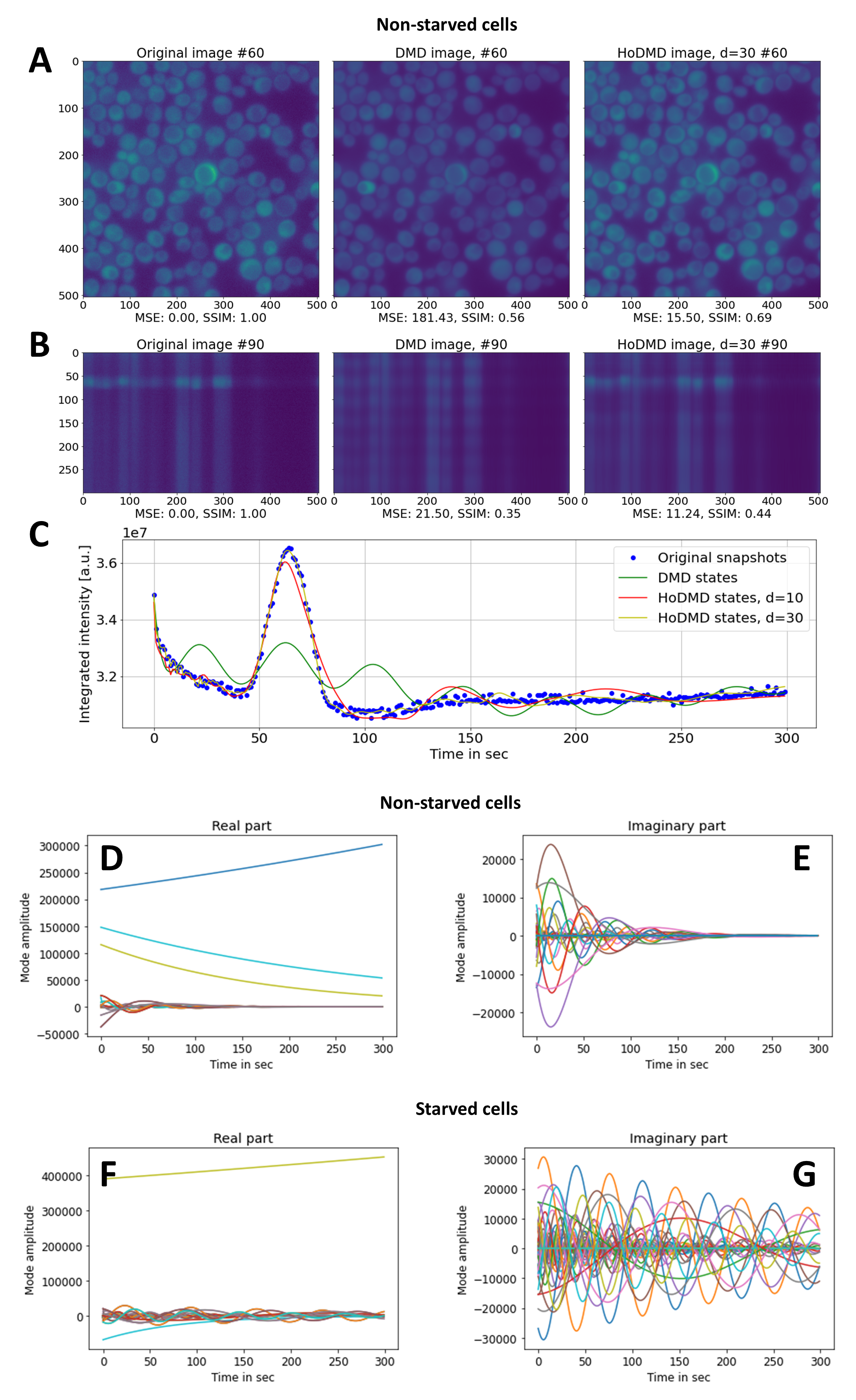** | **Figure S7. HoDMD on image stacks of autofluorescence dynamics in non-starved yeast cells.** Cells were imaged on a wide field microscope every second, and the resulting image stacks were analyzed by standard DMD (‘DMD’) or by higher-order DMD (‘HoDMD’). A, xy-view of one selected frame (frame 60); B, xz-profile along one selected line (line 90) for all three conditions. C, integrated intensity for the original snapshots (blue dots), the DMD reconstruction (green line) and the HoDMD reconstruction with either a delay of *d* = 10 (red line) or *d* = 30 (yellow line). Dynamics of the modes identified by HoDMD for this data, the non-starved cells, is shown in D (real part) and E (imaginary part). |
| --- | --- |
| For comparison, the dynamics of the modes identified by HoDMD for starved cells (compare Fig. 6) are shown in F (real part) and G (imaginary part). | |

| **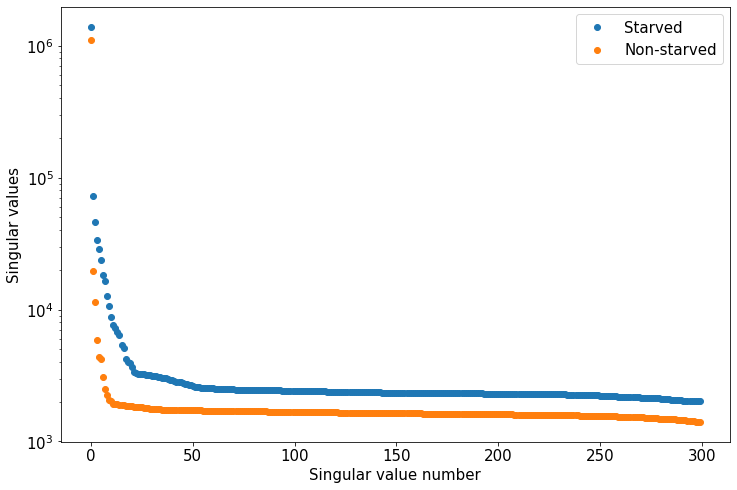** | **Figure S8. Singular value spectrum of image data for starved and non-starved cells.** The singular values were calculated for the experimental data and plotted on a semi-logarithmic scale for starved (blue dots) and non-starved cells (orange dots). |
| --- | --- |

**Additional tests for assessment of forecasting simulated and experimental time series by DMD**

To assess the prediction accuracy of DMD in the critical parameter range, in which the system forms a limit cycle, the simulations were repeated for a mean glucose influx of v = 9.5 mM/min with σ = 0.05 mM/min. For the resulting sustained oscillations, DMD’s ability to predict future time points is worse but can be improved by increasing the delay embedding dimension, *d* (Fig. S9E-H).

When only the first 100 data points of the experimental time series were in the training set, DMD predictions were very poor with very negative R^2^-values (< 400) indicating that the residual error is much larger than the data variance itself. Also for 200-400 data points out of the 600 total in the training set, the R^2^-values were negative, but they approached R^2^=0, which corresponds to the model being as good as the data mean for prediction. With respect to the training data, using a delay embedding of *d* = 25 corresponding to 25% of the truncated data for the 100 data points does not fit the training data well and fails to predict future time points (Fig. S10A). However, when the delay step is increased to 50% of the data points, the model achieves a near-perfect reconstruction of the training data, even for this low number of seen data points, while it still fails on the test data (Fig. S10B). Conversely, increasing the delay step further to 75% of the 100 data points in the training set results in a poor fit once again in both, the training and test data (Fig. S10C). This trend is also seen for truncating the time series after 400 time points (Fig. S10D-F) and is, in fact, consistent across all truncation scenarios (see Fig. S10G-H). The quality of the reconstruction and prediction of the time series by DMD is quantified in the R^2^ plot shown in Fig. S10G for all data (i.e. training and unseen test data) and in Fig. S10H for the training data only.

| 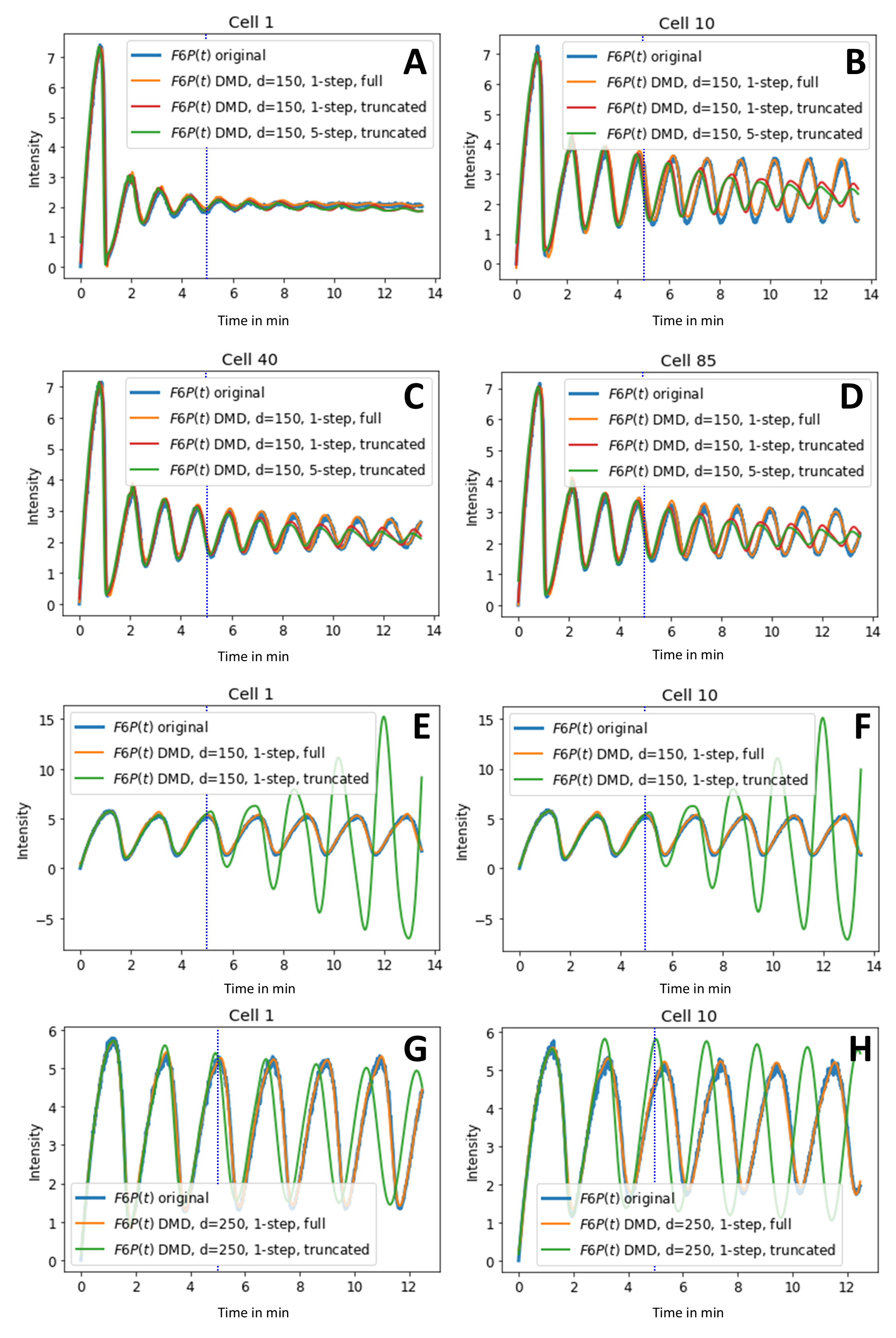 |
| --- |
| **Figure S9. Prediction capability of DMD with delay embedding on simulated time courses.** The ODE system of Eqs. S1 and S2 was simulated using 100 randomly generated values of glucose influx, v, as described in the main text, either with mean influx being 14.5 mM/min to get damped oscillations (A-D) or 9.5 mM/min to get sustained oscillations (E-H). Time courses for F6P are shown for selected cells as blue lines. DMD reconstructions with delay, *d* = 150 (A-F) or *d* = 250 (G-H) are shown. Color coding of the reconstructions is indicated in the legend of each panel. The vertical blue line shows the time point of truncation, left of which was used for training and right of which is the model prediction. |

The reconstruction performance is clearly highest for 50% delay embedding, and the difference to 25 and 75% delay embedding grows, the fewer data points enter the training (Fig. S10H). Down-sampling the data affects reconstruction more than forecasting (Fig. S11).

| **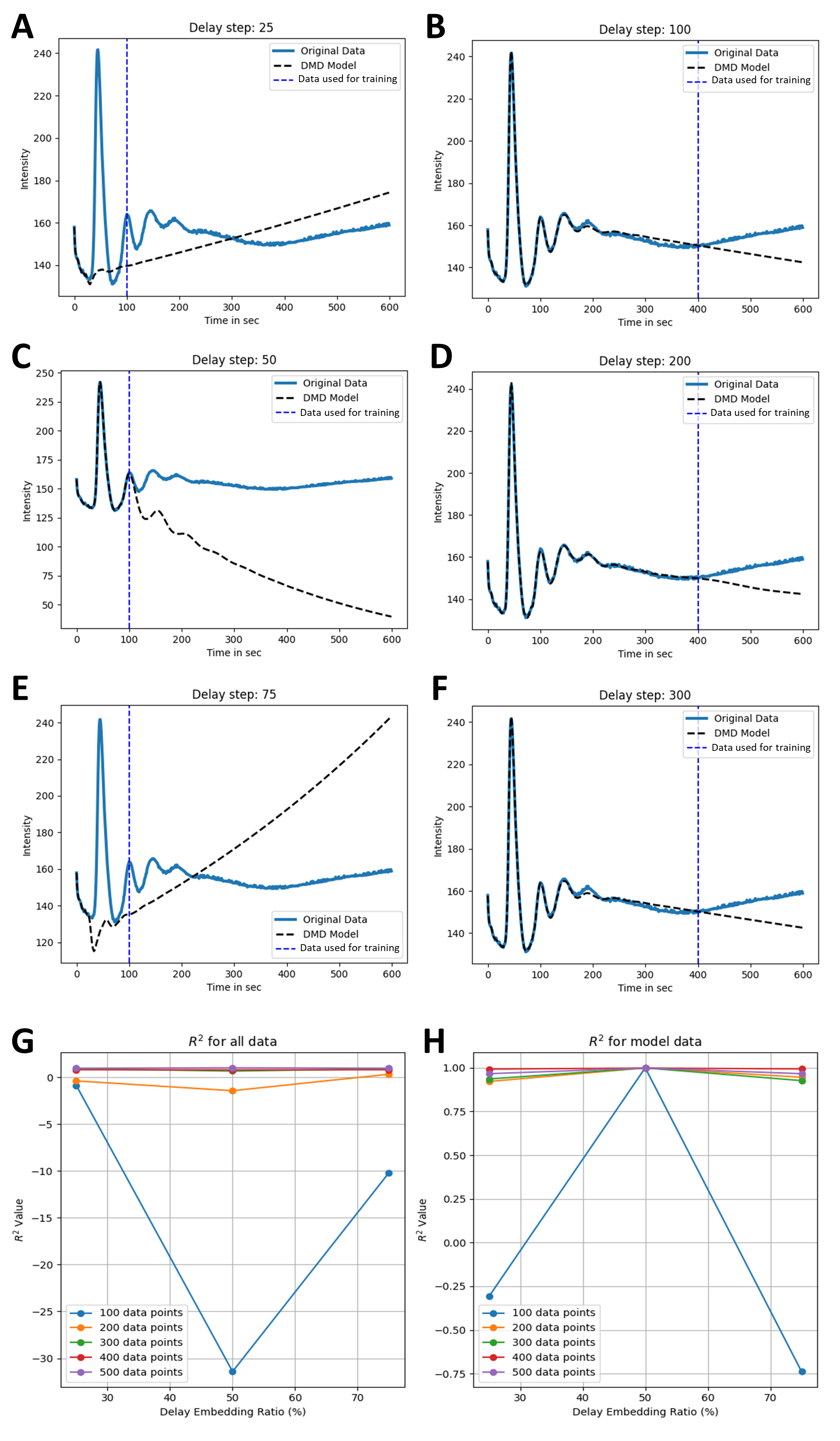** | **Figure S10. Prediction of experimental time courses using DMD with TDE.** Experimental time courses of NAD(P)H fluctuations recorded in response to addition of 15 mM glucose were averaged for all cells in a field (blue lines in A-F, labeled ‘Original data’) and reconstructed using DMD with TDE (black dashed lines in A-F, labeled ‘DMD model’). Training data started at t=0, and its length was varied with 100 time points in A, C and E, and 400 time points in B, D and F, as indicated by the dashed blue line in panel A-F (labeled ‘Data used for training’). G and H show the R^2^-value calculated between either all data (G) or only training data (H) and the corresponding DMD reconstruction/prediction as function of delay, d, for different lengths of the training data. See text for further explanations. |
| --- | --- |

| **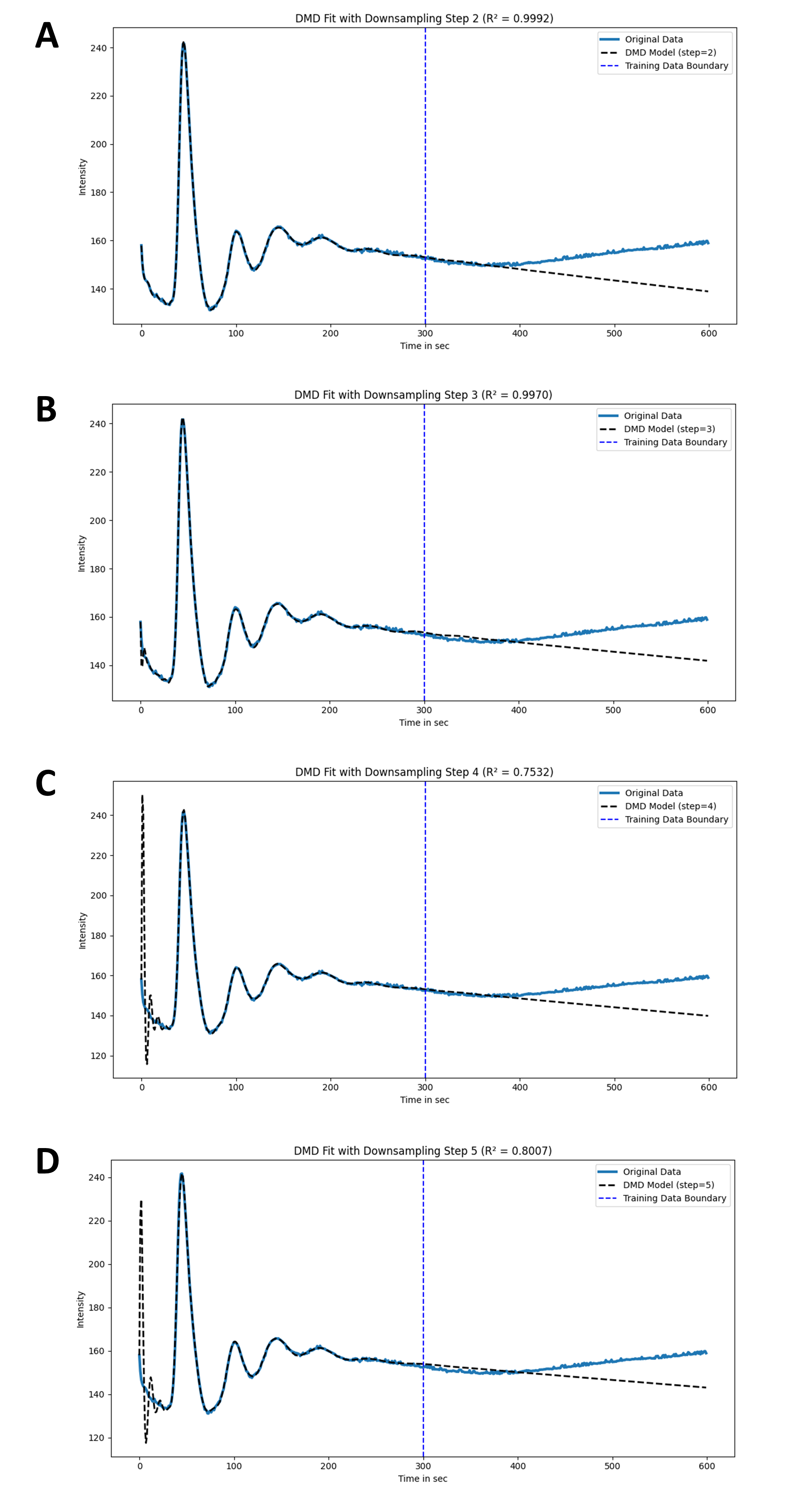** | **Figure S11. Prediction of experimental time courses with missing values.** Experimental time courses of NAD(P)H fluctuations recorded in response to addition of 15 mM glucose were averaged for all cells in a field (blue lines in A-D, labeled ‘Original data’) and reconstructed using DMD with TDE (black dashed lines in A-D, labeled ‘DMD model’). Training data started at t=0, and its length was varied with 300 time points). In contrast to Fig. S10, the training data was down-sampled by removing every other point (A). In B to D, the down-sampling was extended with the training set containing not 150 points, as in A but 100 points (‘Step 3’, B), 75 points (‘Step 4’, C) or even only 40 data points (‘Step 5’, D). At least every third point of the original data is needed for achieving faithful reconstruction. This corresponds to a sampling interval of Δt=3s. Interestingly, the ability to forecast time points is only little affected by down-sampling of this data set. |
| --- | --- |

| **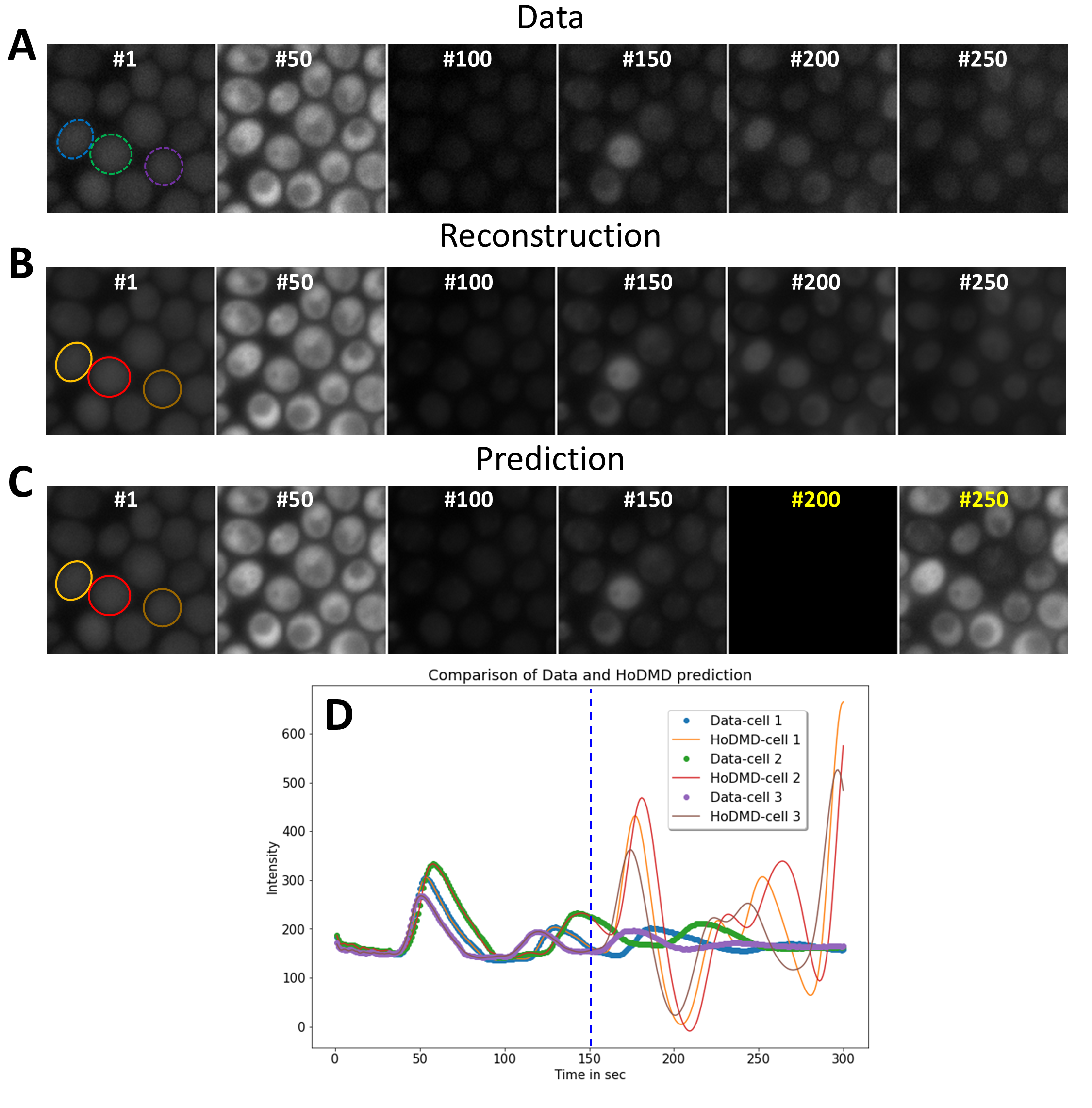** |
| --- |
| **Figure S12. Prediction of unseen images of yeast oscillations by HoDMD.** Image stacks of starved cells were analyzed by HoDMD, and a cropped region of the entire image data is shown (compare Fig. 6D-F). A-C, montage of every 50^th^ frame, starting from the first frame (#1) for the original data, A; the reconstruction, B; and the prediction; C. For B, the training data (white frame numbers) comprised the entire data set, while for panel C, the training data is the first 150 frames, while the last 150 frames are test data for predictions (yellow frame numbers). D, mean intensity profiles for the three cells outlined in A-C (cell 1, yellow; cell 2, red; cell 3, beige) with the blue dashed line indicating the border between training (left) and test data (right). Clearly, the prediction deviates rapidly from the data. |

**Comparison of forecasting potential of DMD with that of recurrent neural networks**

LSTMs are designed to model complex statistical dependencies in the time series data, making them well-suited to model and predict non-linear and non-stationary metabolic oscillations. This is partially a consequence of the use of non-linear activation functions in their architecture but also due to the underlying memory mechanism in such networks, which allows for dynamic adaptation to non-linear variations in the time series data [7]. The idea of including past information into reconstructing the current state and in forecasting future time points is common to both LSTMs and TDE. LSTMs have the ability to adapt their memory length via the gating mechanisms, giving the latter much more flexibility in handling non-stationary and non-linear dynamics. On the other hand, LSTMs learn only an implicit representation of the attractor describing metabolic oscillations, while time-delay methods explicitly reconstruct this attractor. Based on these differences we expect that an LSTM is much more powerful than DMD to predict future time points of the metabolic oscillations. To evaluate the performance of the LSTM model, the training dataset was prepared by sequencing the data into overlapping segments, akin to delay embedding. For example, one segment might include elements 0 to 20, while the subsequent segment spans elements 1 to 21, and so on. This approach ensures the preservation of temporal dependencies within the data. Once the model was trained, predictions for the remainder of the dataset were conducted. Starting with the last value of the training set, the model was used to predict the subsequent value. This predicted value was then iteratively fed back into the model to generate the next value, allowing for sequential prediction of the entire dataset. When applying an LSTM with only one layer to the experimental data with a training time of the first 100 snapshots, the ability to predict future time points was poor and only slightly increased by extending the sequence length (Fig. S13A and B). Including more layers increased the prediction accuracy, but only up to 6 layers, beyond which the R^2^ became worse again, likely due to overfitting (Fig. S13C-L and Fig. S14). Taken together, we found that the performance of both methods, DMD with TDE and LSTM is comparable and in general rather poor, likely because both methods are equally challenged by the non-stationarity of the experimental data, in which no repeating pattern can be predicted.

| **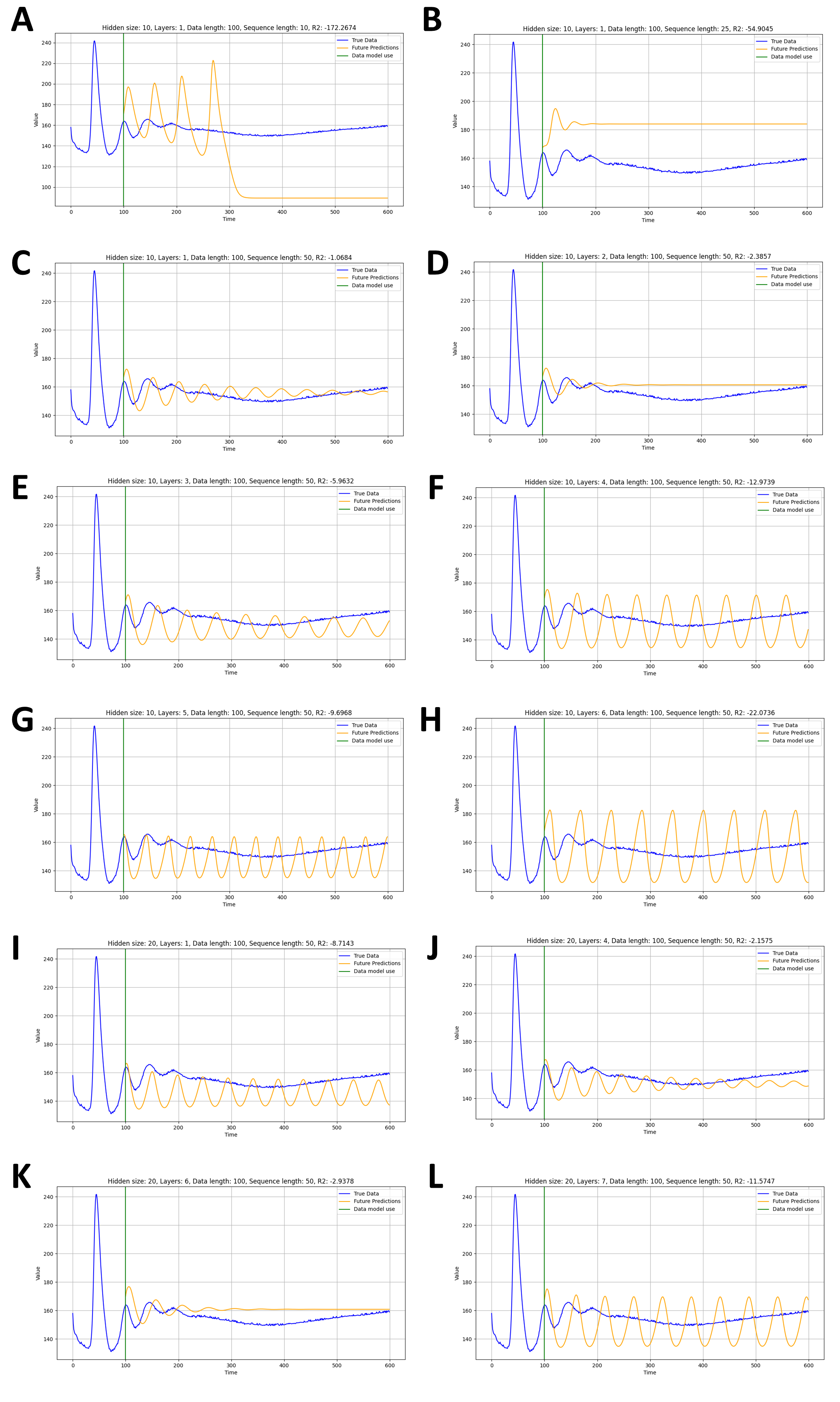** | **Figure S13. Prediction of experimental time courses with LSTM network.** A LSTM was trained on the first 100 data points of the experimental autofluorescence time series (blue lines) to predict subsequent data points (yellow lines). Several network configurations were tested including the number of layers and nodes as well as the percentage of training data included the sequence for training. Parameter combinations are given on top of each panel. The green line indicates the border between training data (0-100) and test data (101-600). See main text for further information. |
| --- | --- |

| **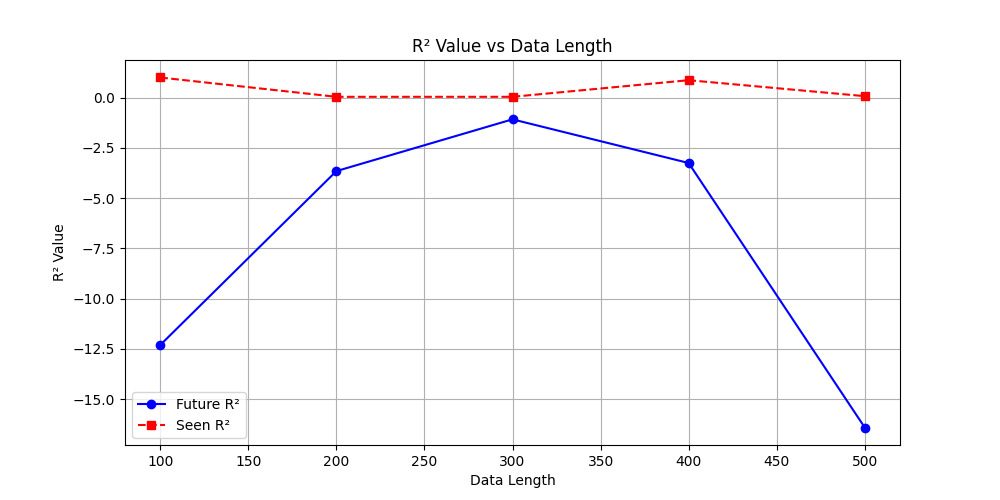** |
| --- |
| **Figure S14. LSTM performance as funcion of size of trainings data.** The LSTM network containing 4 hidden layers and 40 nodes with a sequence length of 50% of the data was trained for 200 epochs with the indicated length of training data, shown on the x-axis. The R^2^-value was calculated separately for the performance on the training data (‘Seen R^2^’, red) and the test data (Future R^2^’, blue). |

| 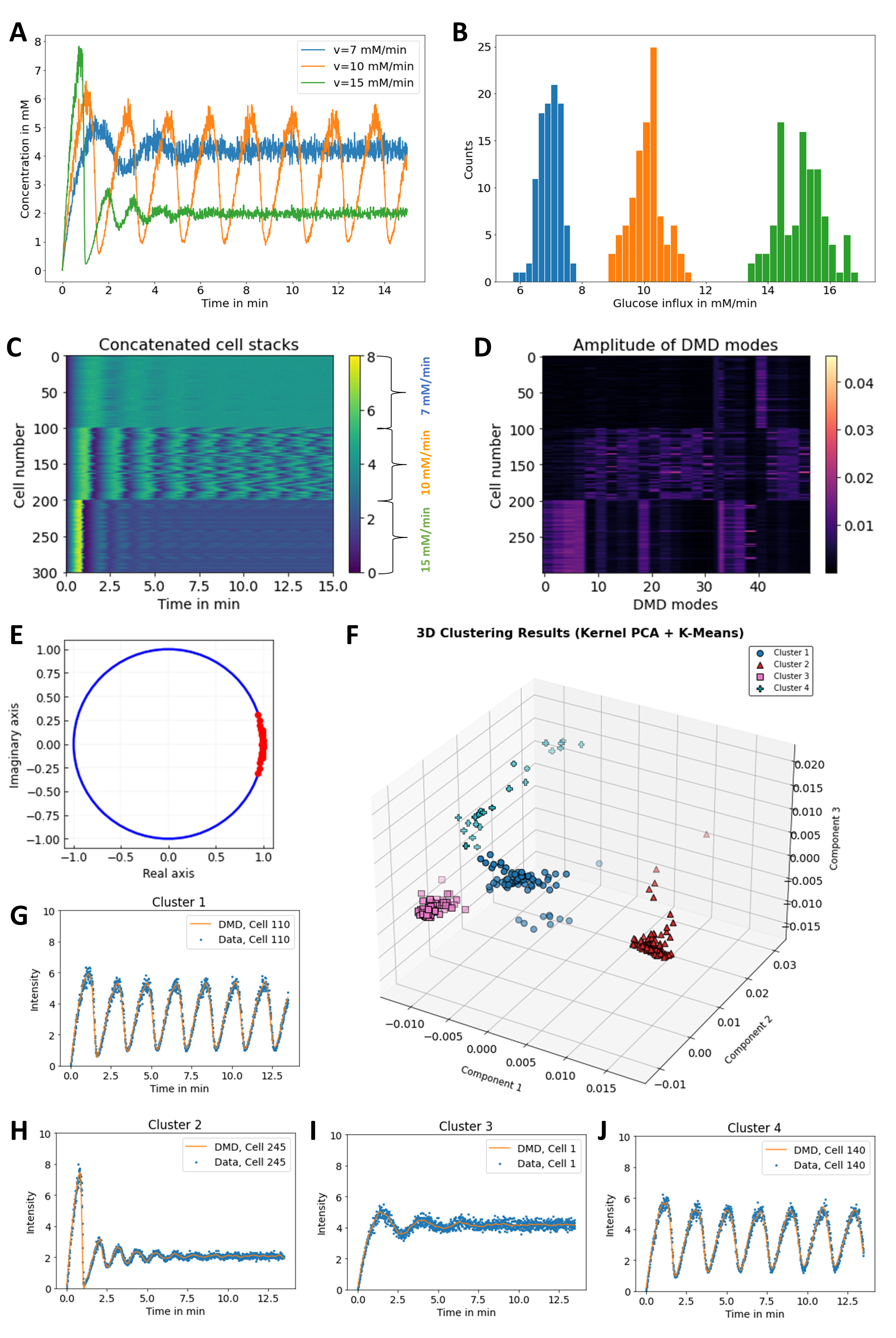 | **Figure S15. Classification of simulated oscillations.** The glycoysis model was simulated with three different influx rates, resulting in very different oscillation behavior, A. To emulate cell-to-cell heterogeneity for each case, three populations of influx values were generated, B. They were used to simulate 100 cells for each condition, and those time traces were concatenated into one matrix, C. DMD with d=100 and rank, r=50 was used to extract 50 modes. |
| --- | --- |
| These DMD modes were used to calculate 50 amplitudes for each cell, D, which were classified by first applying kernel PCA followed by k-means clustering, giving four distinct clusters, F. G-J, shows example trajectories for each cluster (blue dots, data; orange line, DMD reconstruction). | |

**Simulation of calcium oscillations and their reconstruction by DMD**

Calcium oscillations were simulated as described in Materials and methods based on the study by Lavrentovich and Hemkin (2008) [8]. This model is based on the mechanism of calcium-induced calcium release from the endoplasmic reticulum (ER) by inositol-3-phosphate- (IP3) sensitive calcium channels. The model described the dynamics of calcium in the cytosol and the ER as well as the dynamics of IP3. Here, we have only analyzed the cytosolic calcium pool. To mimic cell-to-cell heterogeneity, the activity of the SERCA pump, which transports calcium back into the ER and is an important control point of calcium homeostasis, was stochastically varied. As shown in Fig. S16 below, DMD with delay embedding can reconstruct this data very well, as long as the embedding dimension is very high. The latter is needed, because calcium spikes are the result of multiple non-linearities due to feedback coupling in the pathway components. DMD is also much better suited for denoising of time traces than FFT-based filtering (Fig. S16E). The latter gave a poor data reconstruction.

| 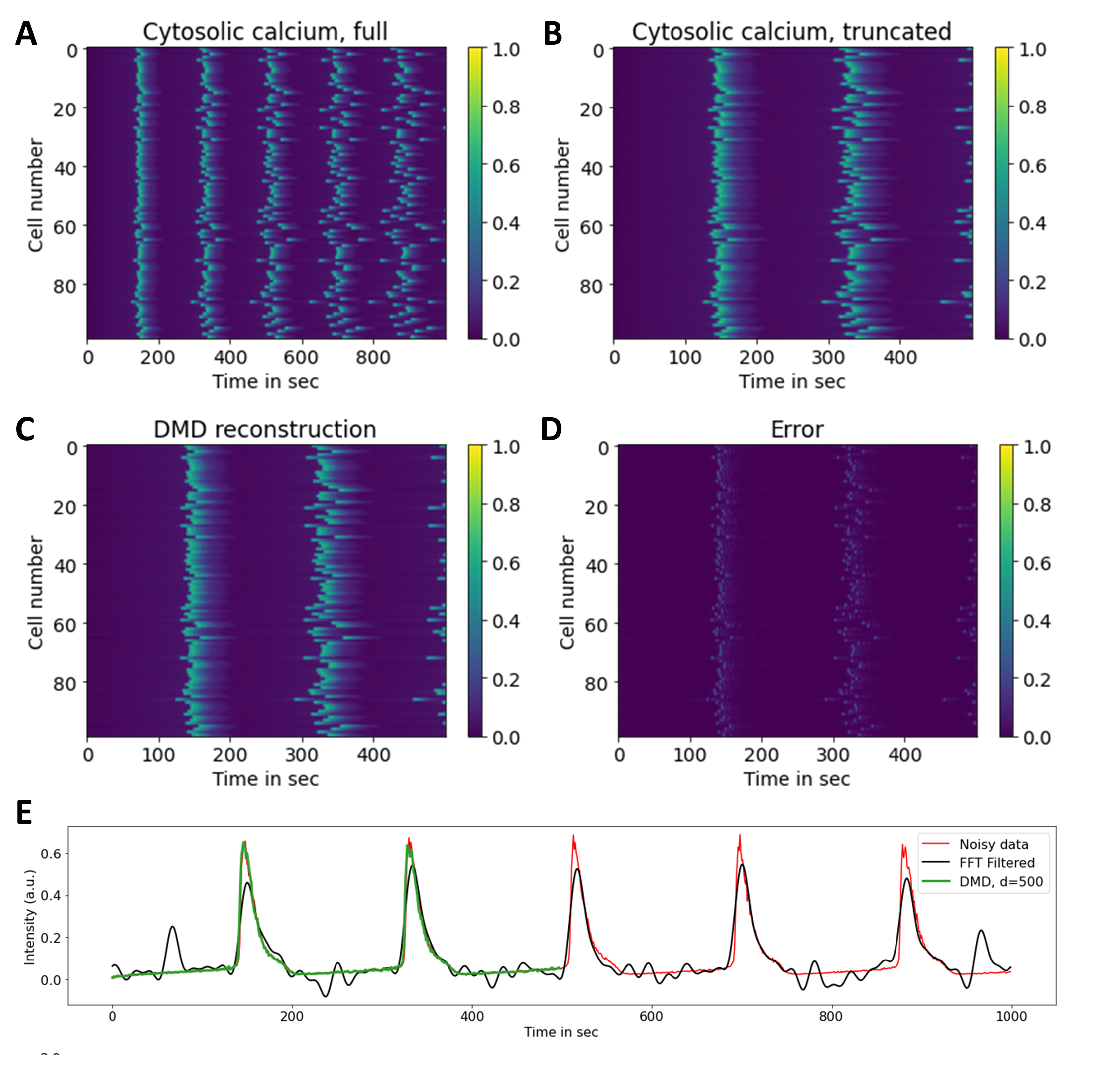 | **Fig. S16. DMD analysis of simulated calcium oscillations.** A, simulation output for 100 cell traces with stochastically varying SERCA activity. B, truncated data set used for DMD upon delay embedding. C, DMD reconstruction and its error compared to the input data, D. An example time trace (red line) is shown together with the DMD (green line) and FFT reconstruction (black line) in panel E. Only the first 500 time points were analyzed by DMD due to the time delays |
| --- | --- |
|  | |
| . | |

**Table S1. Analysis of cell clustering in NPC compared to control cells**. Percentage of cells in a given cluster is calculated. The total number of cells was n=139 (Cluster 1), n=14 (Cluster 2), n=111 (Cluster 3) and n=36 (Cluster 4).

| **Cell type** | **Cluster 1** | **Cluster 2** | **Cluster 3** | **Cluster 4** |
| --- | --- | --- | --- | --- |
| *ncr1Δ* cells | 51.1% | 57.1% | 0.0% | 27.8% |
| *npc2Δ* cells | 48.9% | 35.7% | 1.8% | 47.2% |
| *wt* cells | 0.0% | 7.2% | 98.2% | 25.0% |

**Additional References**

[1] M.F. Madsen, S. Dano, P.G. Sorensen, On the mechanisms of glycolytic oscillations in yeast, Febs Journal, 272 (2005) 2648-2660.

[2] J. Wolf, J. Passarge, O.J. Somsen, J.L. Snoep, R. Heinrich, H.V. Westerhoff, Transduction of intracellular and intercellular dynamics in yeast glycolytic oscillations, Biophysical journal, 78 (2000) 1145-1153.

[3] M.J.B. Hauser, Synchronisation of glycolytic activity in yeast cells, Curr Genet, 68 (2022) 69-81.

[4] D.D. van Niekerk, M. van Wyk, T. Kouril, J.L. Snoep, Kinetic modelling of glycolytic oscillations, Essays in Biochemistry, 68 (2024) 15-25.

[5] R. Heinrich, and Schuster, S., The regulation of cellular processes, Chapman & Hall, New York, USA, (1996) 123-134.

[6] B.P. Ingalls, Mathematical modeling in systems biology: an introduction., MIT press, Place Published, 2013.

[7] S. Hochreiter, J. Schmidhuber, Long short-term memory, Neural Comp., 9 (1997) 1735-1780.

[8] M. Lavrentovich, S. Hemkin, A mathematical model of spontaneous calcium(II) oscillations in astrocytes, J Theor Biol, 251 (2008) 553-560.
