# Supplementary material for: Dynamic mode decomposition for analysis and prediction of metabolic oscillations from time-lapse imaging of cellular autofluorescence
Source: Sci Rep. 2025 Jul 2;15:23489. doi: 10.1038/s41598-025-07255-4 (PMC12222966; doi:10.1038/s41598-025-07255-4)
Supplement: Supplementary file 2 — Supplementary Material 2 [file 41598_2025_7255_MOESM2_ESM.pdf]

Appendix

Stability analysis of steady state of the glycolysis oscillation model

The steady state concentrations of F6P, $\bar{S_1}$, and of F16BP, $\bar{S_2}$, read:

$$\bar{S_1} = \frac{v_0}{k_1 \cdot \left(1 + \left(\frac{v_0}{k_2 \cdot K}\right)^n\right)} \quad \text{(A1)}$$

$$\bar{S_2} = \frac{v_0}{k_2} \quad \text{(A2)}$$

The stability of the steady state can be determined from the Jacobian matrix, which approximates the non-linear dynamics by the first term of a Taylor-expansion according to:

$$J = \begin{pmatrix} \frac{\partial f_1}{\partial S_1} & \frac{\partial f_1}{\partial S_2} \\ \frac{\partial f_2}{\partial S_1} & \frac{\partial f_2}{\partial S_2} \end{pmatrix} \quad \text{(A3)}$$

Here, f_1 and f_2 correspond to the two differential equations given in Eq. S1A, B, and the four matrix entries read:

$$\frac{\partial f_1}{\partial S_1} = a_{11} = -k_1 \cdot \left(1 + \left(\frac{\bar{S_2}}{K}\right)^n\right) \quad \text{(A4)}$$

$$\frac{\partial f_1}{\partial S_2} = a_{12} = -\frac{n \cdot k_1 \cdot \bar{S_1} \cdot \left(\frac{\bar{S_2}}{K}\right)^n}{\bar{S_2}} \quad \text{(A5)}$$

$$\frac{\partial f_2}{\partial S_1} = a_{21} = k_1 \cdot \left(1 + \left(\frac{\bar{S_2}}{K}\right)^n\right) \quad \text{(A6)}$$

$$\frac{\partial f_2}{\partial S_2} = a_{22} = \frac{n \cdot k_1 \cdot \bar{S_1} \cdot \left(\frac{\bar{S_2}}{K}\right)^n}{\bar{S_2}} - k_2 \quad \text{(A7)}$$

The eigenvalues of the Jacobian matrix read:

$$\lambda_1 = \frac{a_{11}}{2} + \frac{a_{22}}{2} - \frac{\sqrt{a_{11}^2 - 2a_{11} \cdot a_{22} + 4a_{12} \cdot a_{21} + a_{22}^2}}{2} \quad \text{(A8)}$$

$$\lambda_2 = \frac{a_{11}}{2} + \frac{a_{22}}{2} + \frac{\sqrt{a_{11}^2 - 2a_{11} \cdot a_{22} + 4a_{12} \cdot a_{21} + a_{22}^2}}{2} \quad \text{(A9)}$$

Since all parameters (i.e., inflow rate, v_0, rate constants, k_1 and k_2, equilibrium constant, K, and Hill coefficient, n) are larger than zero, one can immediately see that always $a_{11} < 0$ and $a_{12} < 0$ and $a_{21} > 0$. The last element, a_{22}, can be positive or negative, depending on the value of the rate constant k_2 compared to the first term in Eq. A7. The eigenvalues can be real or complex, as further explained in the main text. The trace of the Jacobian is

$$Tr(A) = \frac{n \cdot k_1 \cdot \overline{S_1} \cdot \left(\frac{\overline{S_2}}{K}\right)^n}{\overline{S_2}} - k_1 \cdot \left(1 + \left(\frac{\overline{S_2}}{K}\right)^n\right) - k_2 \qquad \text{(A10)}$$

And its determinant reads

$$|A| = k_1 \cdot k_2 \cdot \left(\frac{\overline{S_2}}{K}\right)^n + k_1 \cdot k_2 \qquad \text{(A11)}$$

One sees that $|A|>0$, for all physiologically relevant parameter choices. Together with the eigenvalues, the trace and determinant, the roots of the characteristic equation of the Jacobian can be calculated which, following the Hurwitz criterion, inform about the stability of the steady state (see main text).
